# Fatigue interventions in long term, physical health conditions: A scoping review of systematic reviews

**DOI:** 10.1371/journal.pone.0203367

**Published:** 2018-10-12

**Authors:** Katrin Hulme, Reza Safari, Sarah Thomas, Tom Mercer, Claire White, Marietta Van der Linden, Rona Moss-Morris

**Affiliations:** 1 Health Psychology Section, Institute of Psychiatry, Psychology and Neuroscience, King’s College London, London, United Kingdom; 2 Health Psychology Department, Staffordshire University, Stoke-on-Trent, United Kingdom; 3 Health and Social Care Research Centre, University of Derby, Derby, United Kingdom; 4 Faculty of Health and Social Sciences, Bournemouth University, Bournemouth, United Kingdom; 5 Centre for Health, Activity and Rehabilitation Research, Queen Margaret University, Edinburgh, United Kingdom; 6 Division of Health & Social Care Research, Faculty of Life Sciences & Medicine, King’s College London, London, United Kingdom; Universitat Wien, AUSTRIA

## Abstract

**Objective:**

Fatigue is prominent across many long term physical health conditions. This scoping review aimed to map the fatigue intervention literature, to ascertain if certain interventions may be effective across conditions, and if novel interventions tested in specific long term conditions may be promising for other conditions.

**Methods:**

Scoping review methodological frameworks were used. Electronic bibliographic databases were searched (inception to November 2016) for systematic reviews of fatigue interventions in long term conditions. Inclusion criteria were: long term physical health condition; review focus on fatigue management; objective and systematic review process; primary review outcome is fatigue. Articles focussing on surgical interventions or treatments thought to trigger fatigue were excluded. A narrative synthesis was performed.

**Results:**

Of 115 full texts screened, 52 reviews were included. Interventions were categorised as pharmacological and non-pharmacological (exercise, psychological/behavioural and complementary medicine). Pharmacological interventions did not consistently demonstrate benefit, except for anti-TNFs and methylphenidate which may be effective at reducing fatigue. Non-pharmacological interventions such as graded exercise and fatigue-specific psychological interventions may be effective, but heterogeneous intervention components limit conclusions. ‘Complementary medicine’ interventions (e.g. Chinese herbal medicines) showed promise, but the possibility of publication bias must be considered.

**Conclusions:**

Further research is necessary to inform clinical practice. The reported effectiveness of some interventions across inflammatory health conditions, such as anti-TNFs, aerobic exercise, and psychologically based approaches such as CBT, highlights a potential transdiagnostic avenue for fatigue management. More novel strategies that may be worth exploring include expressive writing and mindfulness, although the mechanisms for these in relation to fatigue are unclear. More work is needed to identify transdiagnostic mechanisms of fatigue and to design interventions based on these.

## Introduction

Fatigue can be described as a lack of energy, feeling of exhaustion or overwhelming sense of tiredness (either physical or mental), that is not relieved by rest and is a common and debilitating symptom in many long-term, physical health conditions (LTCs) [1–4]. LTCs are, defined as a physical health conditions that require on-going management over a period of years [5], for example liver disease, HIV, rheumatoid arthritis (RA) and multiple sclerosis (MS)[6,7]. Recently there has been a move towards conceptualising fatigue as having commonalities across conditions and identifying person-specific (rather than solely illness-specific) factors across domains (i.e. behavioural, cognitive, physiological, social and emotional) [8,9] in a more transdiagnostic approach to management.

A recent scoping review of systematic reviews investigated interventions for fatigue in adults with advanced progressive illness. Authors concluded there is a lack of robust evidence available in support of intervention effectiveness [10]. However, only Cochrane reviews were searched, and only six conditions included under ‘advanced progressive illness’ (cancer, motor neuron disease, chronic obstructive pulmonary disease, cystic fibrosis, HIV/AIDS and MS). The search for relevant articles is likely to have been limited and insights missed from other conditions where fatigue is prominent, e.g. chronic fatigue syndrome and rheumatoid arthritis [11,12]. Additional systematic reviews have also been conducted since, so an updated and more inclusive scoping review may provide more in-depth insights of clinical relevance.

Scoping reviews are a relatively new approach to collating health research evidence. They aim to map the existing literature in a field of interest and can be of particular use for topics that have not been extensively reviewed or are complex in nature [13]. They are becoming increasingly popular due to their ability to map key concepts, sources of evidence and types of evidence underpinning a research area [13]. They are valuable for comparing reviews of fatigue interventions across health conditions, where different study designs and interventions may be of interest [14].

The aims of this scoping review are:

1. Map the range of reviews conducted on interventions used to treat, reduce or manage fatigue in LTCs, defined as a long-term, physical health condition
2. Identify commonalities between interventions used across conditions and whether these are effective, which may highlight transdiagnostic aspects of fatigue management.
3. Identify novel interventions trialled in any single condition, which may be promising in the treatment of fatigue in other health conditions.

## Methods

### Protocol

A protocol was developed prior to conducting this review, based upon detailed methodology outlined in the Joanna Briggs Institute (JBI) Reviewers’ Manual [15] and the framework proposed by Arksey & Malley [14] and updated by Levac et al. [16]. This framework consists of six stages: (1) identifying the research question, (2) identifying relevant studies, (3) selecting studies, (4) charting the data, (5) collating, summarising and reporting the results, and (6) consultation. The scoping review process is an iterative one, particularly during the study selection phase [16]. During full text screening, the inclusion and exclusion criteria were refined for clarity and specificity to the review’s objectives after discussion amongst the research team. In line with the published guidelines for scoping reviews [15] and the aims of mapping rather than rating the existing literature, we did not conduct quality assessment.

### Stage 1: Identifying the research question

The specific questions guiding this scoping review were: i) What fatigue interventions are used to treat, reduce or manage fatigue in LTCs, defined as a long-term, physical health condition?? ii) What interventions are commonly used across conditions, and which are consistently reported as effective? iii) Which novel interventions from individual LTCs may be promising?

### Stage 2: Identifying relevant studies

#### Search strategy

Comprehensive and systematic electronic searches were designed, refined and conducted in consultation with an information specialist, combining MeSH terms and keywords relating to ‘fatigue intervention’ and ‘review’ (full search strategies can be found in Appendix A). Search strategies were designed to maximise comprehensiveness and sensitivity in the initial search [15], and then increase specificity in the later screening. Therefore, names of individual conditions, as well as the term ‘long term condition’, were not specified in the initial search criteria. This minimised the risk of missing potentially relevant research, either reviews in less well-known LTCs or those in individual conditions not specifying ‘long term condition’ as a key word for searches.

The following databases were searched from their inception to November week 1, 2016: Cochrane Database of Systematic Reviews, PsycINFO; EMBASE; OvidMEDLINE(R); OvidMEDLINE(R) in process and other non-indexed citations; CINAHL; Web of Science; LILACS; PEDro. OpenGrey and Google Scholar were used to search for grey literature, and experts in the field were contacted. Reference lists and citations of included reviews were hand-searched. Only articles in the English language were considered for inclusion.

### Stage 3: Selecting studies

#### Eligibility criteria

Inclusion and exclusion criteria can be found in Table 1. It is recommended that eligibility criteria are defined through an iterative process [15]. Therefore, we refined the inclusion and exclusion criteria during the screening process, in collaboration with an information specialist, to ensure our research aims were accurately met:

1. Some reviews were found to group healthy and LTC populations together during analysis and not clearly describe the different populations, so we refined our initial inclusion criterion of ‘long term physical health conditions’ to ‘*solely* long term physical health conditions, *clearly described’*.
2. Initially our focus was interventions with fatigue as the primary outcome. It became clear that most reviews included studies which had measured fatigue as a primary or secondary outcome. To maintain breadth across the field of research, we shifted the focus to defining the primary outcome of the systematic review.
3. In addition to point 2, we specified that fatigue management was the focus of review as some reviews include measures of fatigue but focus on reviewing ways of assessing fatigue or documenting fatigue as a side effect of a treatment.
4. The exclusion criterion ‘population undergoing surgical intervention’ was added during the second screening phase, as surgery was deemed to be a permanent, condition specific procedure (where fatigue was not the primary outcome), not of relevance to our aims of identifying transdiagnostic interventions.
5. Similarly, fatigue caused by treatments or medications for specific conditions has a physiological basis that was beyond the scope of this review, thus an exclusion criterion was defined during the second phase of screening. These iterations were defined after discussion amongst the research team.

**Table 1:** Inclusion and exclusion criteria.

Inclusion	Exclusion
Solely long term physical health condition, clearly described.	Population undergoing surgical intervention.
Fatigue management is a main focus of review.	Focus on a population undergoing treatment causing fatigue.
Objective and systematic review process; 2+ databases searched, screening process/criteria for inclusion.	Article is a previous version of a more recent, updated review.
Primary outcome of review is fatigue (i.e. studies included target or evaluate fatigue).	Article not in English.

#### Types of studies

We initially searched for reviews of fatigue interventions, including (but not limited to) systematic reviews, meta-analyses, narrative synthesis, literature reviews and scoping reviews, to ensure comprehensive identification of potentially relevant articles. Inclusion criteria were employed to select reviews which had systematically searched for papers, to identify the highest order of evidence. We defined ‘systematically conducted’ as employing a systematic and objective search strategy, using inclusion criteria and searching two or more databases.

#### Population

The population of interest was ‘long term health condition’, defined as a long-term, physical health problem.

#### Concept

Interventions could include (but were not limited to) pharmacological, psychological, educational, behavioural and exercise methods. Initially, the focus was interventions with a primary aim of treating fatigue but it became clear that relevant reviews often included studies which had measured fatigue as both a primary or secondary outcome. Rather than specifying the outcomes of individual studies, we specified that a primary outcome and focus of the review had to be fatigue.

Interventions for fatigue due to treatment, as opposed to the health condition, were beyond the scope of this review. As cancer treatment is known to be associated with fatigue after treatment and reviews did not typically distinguish between disease-related and treatment-related fatigue, cancer-related fatigue was not included in analyses.

#### Context

No limitations were set regarding the context of reviews.

#### Screening

Titles and abstracts of identified articles were screened by KH. Full texts of potentially relevant articles were then screened by KH against the eligibility criteria, 10% of which were quality checked/screened by ST. Any articles where inclusion was unclear were cross-checked and discussed between KH and ST.

In the absence of full texts of abstracts or review protocols, authors were emailed to ask for the full review article. In the case of abstracts, if the author did not reply but enough information was included for a decision to be made using the eligibility criteria, abstracts were evaluated for inclusion.

### Stage 4: Charting the data

#### Data extraction

Data were extracted using two tables; one for review characteristics and the other for fatigue related findings of each review (see supplementary tables for further details). Data extraction was conducted by KH and RS, with half of full texts assigned to each extractor. Double extraction occurred for the first ten articles. Extraction was discussed and any necessary amendments to extraction table headings were made to maximise clarity and accuracy.

### Stage 5: Collating, summarising and reporting the data

#### Data synthesis

A descriptive numerical summary and tabulation of findings was conducted. Findings were presented in a narrative synthesis, grouped by type of intervention, to identify fatigue interventions common (and beneficial) across LTCs, as well as interventions only implemented in certain physical health conditions.

### Stage 6: Consultation

Experts in the field of fatigue and individual health conditions were consulted during protocol preparation, to help establish inclusion criteria and identify grey literature. Findings were also discussed.

## Results

### Search and selection of included reviews

The database search returned 5,232 records, of which 97 were deemed potentially relevant after removing duplicates and title/abstract screening. Searching additional sources (e.g. references from experts and reference/citation lists) identified 18 further articles of potential relevance. In all, 115 articles were assessed for eligibility.

Of the four authors emailed about conference abstracts, none responded. For protocols, three authors (for four of eight protocols) replied, indicating that reviews were ongoing [17–20].

**Fig 1:**
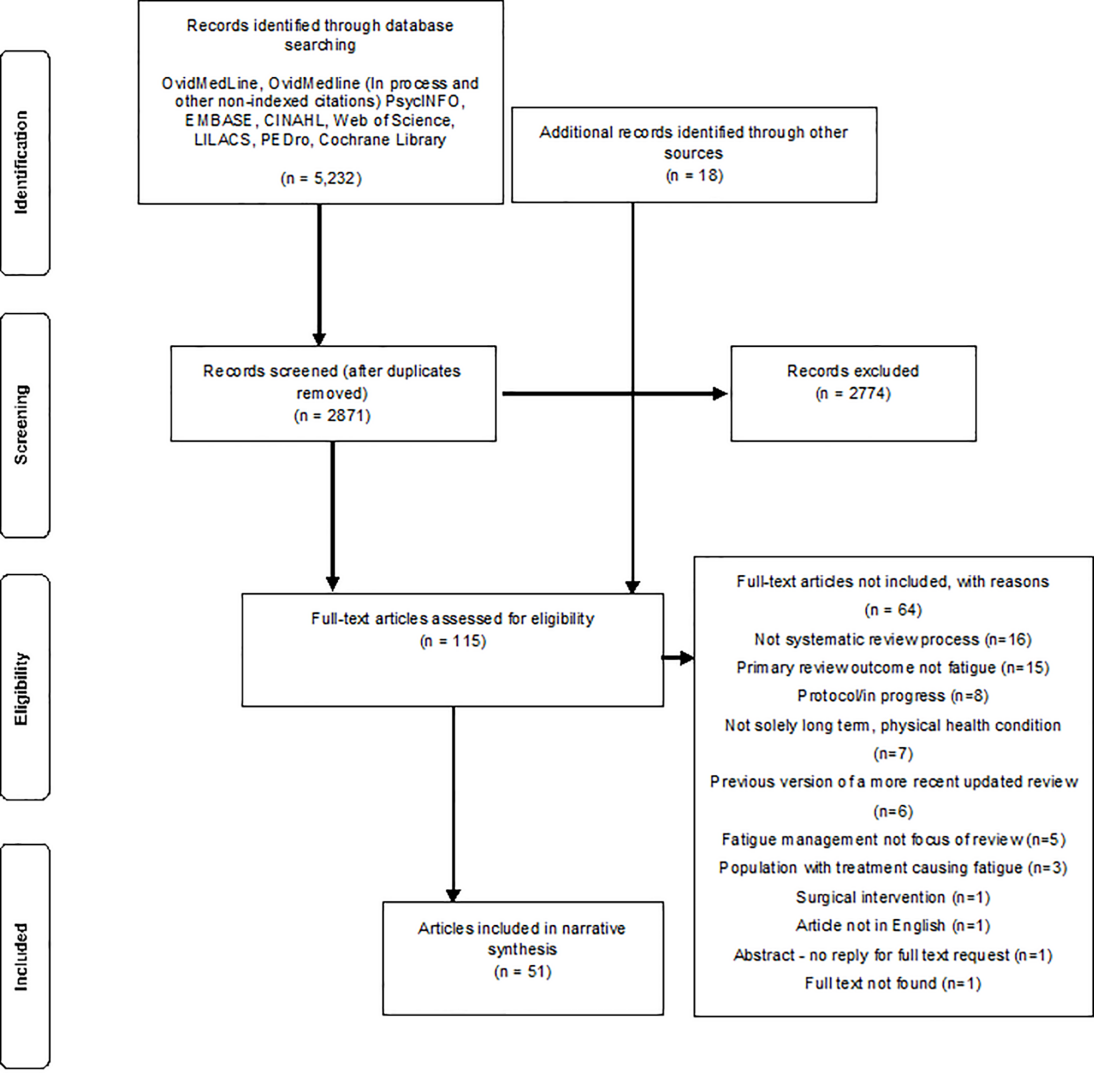
Flow diagram detailing the inclusion/exclusion process.

After full text screening, 51 articles were retained for inclusion in the scoping review, encompassing 52 reviews (Fig 1). Branas et al. [21] included both a scoping and a systematic review in one article so both were included.

### Included reviews

An overview of the included reviews can be seen in Table 2. References for all included reviews can be found in supplementary information (S1 Text). Review characteristics are also outlined in more detail in supplementary information (S1 Table), as are review findings (S2–S5 Tables).

**Table 2:** Overview of reviews.

Review characteristics (N = 52)		Count (%)
Review type	Narrative synthesis	18 (35%)
	Meta-analysis	24 (46%)
	Scoping review	3 (6%)
	Systematic overview (including specific section on fatigue interventions)	4 (7%)
	Conference proceedings—abstract	3 (6%)
Year of publication	No date	2 (4%)
	Pre-2005	2 (4%)
	2006–2011	15 (29%)
	2012–2015	23 (44%)
	2016	8 (15%)
	2017	2 (4%)
Health condition studied	Multiple Sclerosis	18 (34%)
	Chronic Fatigue Syndrome	8 (15%)
	‘Mixed’ clinical populations	6 (11%)
	Parkinson’s Disease	3 (6%)
	Systemic Lupus Erythematosus	3 (6%)
	Rheumatoid Arthritis	3 (6%)
	End Stage Kidney Disease	2 (4%)
	Inflammatory Bowel Disease	1 (2%)
	Sarcoidosis	1 (2%)
	Traumatic Brain Injury	1 (2%)
	HIV	1 (2%)
	Heart disease	1 (2%)
	Fibromyalgia	1 (2%)
	Sjogren’s Syndrome	1 (2%)
	Peripheral neuropathy	1 (2%)
	Post-stroke	1 (2%)
Review focus	Pharmacological interventions Non-pharmacological interventions Mixture/not specified	10 (19%) 28 (54%) 14 (27%)
Review size	Number of studies included in reviews - Minimum to maximum - Average	0–45 17

The majority of included reviews have been published or conducted in the past five years (n = 33). Twenty two reviews only included randomised controlled trials (RCTs), whereas other reviews included other study designs, such as pretest-posttest, open-label, pilot and crossover trials.

Reviews included a wide range of research articles, ranging from zero (Adams et al. [22] who did not identify any Chinese medicine chronic fatigue syndrome (CFS) intervention papers fulfilling their inclusion criteria) to 45 [23,24]. Reporting of total participant numbers was variable; some reviews omitted this information whilst others provided a range and/or overall total. Of the 38 reviews that stated the total, this ranged from 36 to 14,628 participants. The next largest samples were 4,765, 4,696 and 2,882, with most reviews reporting on fewer than 1,500 participants. The larger participant samples tended to be from reviews including a variety of study designs, investigating several health conditions or reviewing pharmacological interventions.

In line with the aims of this review, findings have been grouped by intervention type; pharmacological and non-pharmacological interventions. Non-pharmacological interventions have been further divided into three broad categories; ‘exercise’, ‘psychological/behavioural’ and ‘complementary medicine’ (Table 3). The descriptors provided for the various interventions grouped under these broad categories are listed in Table 3, which also presents review categories in relation to specified LTCs.

**Table 3:** Overview of fatigue interventions in the included reviews.

Intervention (including common/relevant subtypes)	Health condition															
	CFS	RA	SLE	PD	TBI	IBD	Sarcoidosis	Peripheral neuropathy	Coronary heart disease	HIV	ESKD	Post-stroke	Fibro-myalgia	Sjorgen’s Syndrome	MS	Mixed
**Pharmacological**	1	2	1	3	1	1	1	1		1		1		1	8	2
**Exercise**	4	1	3	3	1	1	1		1		1	1	1		9	2
**Psychological/behavioural**	4	1	3	1	1	1				1	1	1			8	4 (overlap)
1. CBT based	x	x	x	x	x					x	x	x			x	
2. Education		x	x		x							x			x	
3. Management (various)		x	x			x					x				x	
4. Relaxation			x							x						
5. Counselling			x													
6. Mindfulness		x										x			x	x (specific)
7. Expressive writing		x	x													
8. Energy conservation		x													x	
9. Solution focussed therapy						x										
10. Guided imagery																x (specific)
**Complementary medicine**	2	1	2	1	1	1					1	1			3	2 (overlap)
1. Acupuncture (or similar)	x		x								x	x				
2. Herbal medicine	x	x										x				
3. Diet		x	x													
4. Supplements		x	x			x										
5. Phototherapy			x													
6. Pulsed electromagnetic therapy															x	
7. Cooling															x	
8. Electroenc. Biofeedback					x											
9. Cranial electro. stimulation					x											
10. Blue light					x											
11. Reflexology		x														
12. Japanese massage				x												

Note: the numbers given show the number of reviews in each condition that have included interventions classed as pharmacological, exercise, psychological/behavioural and complementary medicine, respectively. (x) denotes more specifically which types of interventions, under the psychological/behavioural and complementary medicine headings, have been investigated in each health condition. Where possible subgroups were provided, however, descriptions of interventions in the exercise category were extremely variable so creating meaningful and accurate subgroups was not possible. Sub-headings are based upon the labels given in reviews.

(CFS; Chronic fatigue syndrome, RA; Rheumatoid arthritis, SLE; Systemic lupus erythematosus, PD; Parkinson’s disease, TBI; Traumatic brain injury, IBD; Inflammatory bowel disease, HIV; Human immunodeficiency virus, ESKD; End stage kidney disease, MS; Multiple sclerosis)

For each intervention type, we first describe the type of interventions used and for which conditions as per aim 1. In line with aim 2 we then describe effectiveness findings, highlighting if these have been shown to be effective across more than one condition (i.e. whether they have potential for transdiagnostic fatigue management). The final section explores novel interventions trialled in any single condition, which may be promising in the treatment of fatigue in other health conditions.

Multiple reviews in the same health condition often included the same intervention studies, so findings are primarily presented in relation to health conditions as a whole, as opposed to each review individually.

### Pharmacological interventions

Pharmacological interventions were considered in 24 reviews, of which 10 focussed specifically on pharmacological agents (see S2 Table).

The most common pharmacological agents in multiple conditions were:

- Psychostimulants; methylphenidate and modafinil (sarcoidosis, Parkinson’s disease (PD), traumatic brain injury (TBI), HIV and MS).
- Anti-TNF biologic agents, such as adalimumab and infliximab (RA, inflammatory bowel disease (IBD), sarcoidosis and Sjogren’s syndrome).
- Dehydroepiandrosterone (DHEA) (systemic lupus erythematosus (SLE), HIV and Sjogren’s syndrome).
- Fluoxetine (HIV, post-stroke and CFS).
- Amantadine (peripheral neuropathy and MS).

In terms of aim 2, the magnitude of effect sizes reported by meta-analyses ranged from 0.07 to 1.23. Anti-TNF biologic agents (for example, adalimumab and infliximab) were consistently reported to be effective in reducing fatigue in RA [25,26], IBD [27], and sarcoidosis [28]. An effect size of 0.43 was reported for biologics in RA, with similar effects for anti-TNFs and non-anti-TNFs [26]. The psychostimulant methylphenidate also showed potential benefit across multiple health conditions, including sarcoidosis, PD, TBI and HIV. Results for the psychostimulant modafinil were inconsistent. It was reported as potentially effective in HIV [29] but findings in MS and TBI were mixed [7, 30–33]. It was reported to be ineffective for PD fatigue [32,34] with a possible trend towards a larger effect in people with depression, than those not depressed [35].

In terms of aim 3, several pharmacological agents were considered in only one or two reviews, but were shown to significantly reduce fatigue in the respective condition of interest: non-anti-TNFs (RA) [25,26]; thiamine (IBD) [27]; N-acetylcysteine and belimumab (SLE) [36]; rasagiline, pergolide mesilate and pramipexole (dopamines) (PD) [35, 37] and prokarin (MS) [30].

### Pharmacological study limitations

Reviews highlighted common limitations of pharmacological studies: small sample sizes, high risk of bias, variety of outcome measures, fatigue included as a secondary outcome, participants not fatigued at baseline, not stating whether change meets minimal clinically important difference, short study duration and inadequate statistical analyses.

### Non-pharmacological interventions

Non-pharmacological interventions were considered in 42 reviews, which we grouped into exercise, psychological/behavioural and ‘complementary medicine’ interventions (S3–S5 Tables).

### Exercise interventions

Twenty-nine reviews addressed the effect of exercise on fatigue. These reviews spanned 12 conditions, as shown in Table 3. Two reviews [38,39] included mixed clinical populations, overlapping with single condition reviews.

Type and mode of exercise interventions varied considerably, both within and between reviews. Across health conditions, most exercise interventions included an aerobic component. Mode of exercise typically included cycling, walking, and swimming; however, climbing, yoga, dancing and aerobics were also featured. Exercise intensity was mentioned in five reviews and was only reported in relation to aerobic exercise. Of the five reviews, two were unable to or did not draw conclusions about exercise intensity [7, 40] (both MS), one merely reported that exercise intensity in studies ranged from low to high [41] (CFS), one reported effective exercise interventions were of moderate intensity [42], (fibromyalgia) and one reported that GET appears to be more effective when started at low levels of intensity [43](CFS).

Resistance training interventions were reported in reviews in sarcoidosis, HIV, CFS, RA and MS, but were the independent focus in only one review in MS [44](Abstract). Most reviews did not compare types of exercise, instead including all under the umbrella term ‘exercise’. Only two reviews (both MS) compared exercise modalities, concluding that a combination of endurance (aerobic) and resistance training may be of most benefit to reduce MS fatigue [23,45], mirroring conclusions drawn by Godhrawala et al. [44]. There was considerable variation within and across reviews in terms of intervention dose frequency and duration. Exercise interventions were reported as typically occurring three times per week, lasting approximately 40 minutes and continuing for around 12 weeks. However, this ranged from exercising one to seven times per week, for 5–60 minutes and continuing for up to six months.

In terms of the effectiveness of these exercise interventions, addressing aims 2 and 3, effect sizes of meta-analyses conducted ranged from 0.36 to 0.68. The considerable variation across reviews in different conditions made it difficult to identify specific details of exercise interventions that were effective across conditions. Within individual conditions, findings suggest graded exercise therapy (GET) could be effective in ameliorating fatigue in CFS [43,46,47]. Physical activity such as yoga, Tai Chi and pool based exercise are reported to be beneficial for RA fatigue [12]. Aerobic exercise was the predominant exercise studied in SLE patients and was reported to reduce fatigue [36, 48, 49]. Findings presented in the following conditions also suggest that exercise may be beneficial in ameliorating fatigue: cardiac rehabilitation in coronary heart disease; exercise advice in IBD; aerobic and resistance training in sarcoidosis; physical activity such as yoga, walking and pool based exercise in fibromyalgia. Reviews also indicate that exercise has an overall moderate effect on fatigue in MS. Findings reported in PD, TBI, post-stroke and ESKD reviews suggest exercise may not be an effective fatigue intervention. However, one should note that the number and quality of studies included in the reviews of PD were limited.

Inconsistencies were reported within reviews as all individual studies did not necessarily find exercise interventions to be beneficial. Six reviews highlighted sub-group differences or moderators as outlined in Table 4. The sub-groups assessed varied, but factors for consideration highlighted by multiple reviews were type of control condition, exercise modality, and whether the study sample was fatigued at baseline.

**Table 4:** Reviews reporting exercise intervention subgroup differences.

Study	Sub-group
Marques et al.[47]—CFS	Analysis: secondary-tertiary interventions, interventions delivered by psychologists or psychotherapists and those providing minimal contact were more effective compared to those in primary care, delivered by other healthcare practitioners (e.g. exercise/physical therapist, nurse, physiologist) and providing intensive contact. Including psychological components and allowing activity flexibility reported as more effective, but with high levels of heterogeneity.
Larun et al. [41]—CFS	Analysis: graded aerobic exercise (versus treatment as usual) was more effective than anaerobic exercise (versus relaxation). No differences between diagnostic criteria or type of control group.
Puetz et al. [50]–coronary heart disease	Analysis: interventions in non-controlled studies were more effective than in controlled studies. No differences by cardiac rehab intervention type (multifactorial versus exercise).
Heine et al. [23]–MS	Analysis: interventions comparing to non-exercise controls (versus exercise controls) were more effective. Intervention type had an effect: endurance and mixed training reduced fatigue, whereas muscle power and task-orientated training did not.
Andreasen et al. [40]—MS	Observation: the majority of studies reporting an effect had fatigued population at baseline, whereas the majority not reporting an effect were those where the study population was not. No difference by exercise modalities. Differing duration, frequency and intensity of exercise, and the use of different fatigue scales was inconclusive. Social interaction and motor mechanisms were broached as potential mechanism for exercise reducing fatigue.
Asano et al. [51]–MS	Secondary reporting: effect sizes for studies including fatigued participants were typically positive and significant, whereas for non-fatigued participants they were negative and non-significant.

### Psychological/Behavioural interventions

Twenty-six reviews addressed the effect of psychological or behavioural interventions on fatigue. These reviews spanned twelve conditions, see Table 3. Four reviews included mixed clinical populations; two reviews combined conditions while investigating general fatigue interventions [38,39], while one focused specifically on mindfulness [52] and one on guided imagery [53].

Cognitive behavioural therapy (CBT) was the most cited intervention, referenced in reviews of fatigue management in all conditions except IBD, either as a standalone treatment or as a basis for treatment, (e.g. CBT based interventions targeting fluid adherence, sleep or physical functioning (ESKD) and cognitive-behavioural stress management (HIV)). Other cross-condition intervention techniques included energy conservation (MS and RA), expressive writing (RA and SLE), (psycho-)education (SLE, TBI, post-stroke, HIV, MS), mindfulness (Post-stroke, TBI, MS– 1 review) and guided imagery (asthma, bronchitis and emphysema, cancer, congestive heart failure, MS, HIV– 1 review). Self-management based interventions were also common, centring around a variety of topics such as fatigue, stress, lifestyle and nutrition (SLE, post-stroke, ESKD, RA, MS, IBD). Finally, other treatment modalities reported included ‘relaxation’, ‘solution-focused therapy’, ‘multi-disciplinary rehabilitation’, ‘counselling’ and ‘community support’. The use of varying terminology and grouping of interventions for overall analysis in reviews must be noted, making accurate comparison of individual intervention mechanisms difficult.

Referring to aim 2, taken together as a broad category, CBT/behavioural interventions were generally reported to be effective in reducing fatigue. Effect sizes reported in meta-analyses ranged from 0.24 to 0.48. CBT, psychoeducation, relaxation, counselling, stress management and expressive writing were reported as effective in SLE [48,49], although evidence was reported as weak and inconsistent by Yuen & Cunningham [36]. Psychosocial interventions, including CBT, expressive writing, mindfulness and group education, were reported to reduce RA fatigue, but this was a grouped analysis so the differential effects of individual modalities was unclear [12]. Again, grouped analyses suggested that psychosocial interventions may be beneficial for ESKD fatigue [54]. Relaxation was shown to significantly reduce fatigue in HIV, however, cognitive-behavioural stress management/psychoeducation was not [29]. In MS, energy conservation and CBT were reported to be effective but long term effects were inconsistent [55,56].

Two reviews focussed on a specific type of intervention, as opposed to a health condition. Mindfulness was reported to have a significant treatment effect on fatigue compared to no treatment or control treatments in post-stroke, TBI and MS [52]. Guided imagery findings were more inconsistent, but it was noted that studies demonstrating a significant improvement were those that had the greatest total duration of exposure, used targeted imagery (based upon condition and outcome) and had over 30 participants [53].

In terms of identifying novel interventions, solution focussed therapy, although only noted in one review (IBD), was reported as a promising intervention.

Psychological/behavioural interventions varied, for example, in duration of sessions, who provided/facilitated the intervention, the length of the intervention, whether it was delivered one-to-one or in a group, mode of delivery (e.g. face-to-face, telephone), and intervention content. Six reviews addressed sub-group differences (Table 5) suggesting that interventions targeting a population fatigued at baseline (compared to non-fatigued) (MS and CFS) and one-to-one interventions (ESKD and mixed in mindfulness) may be more effective. Reviews in PD, TBI and post-stroke reported that there is little evidence for the effectiveness of CBT/behavioural programmes.

**Table 5:** Reviews reporting psychological/behavioural intervention subgroup differences.

Study	Sub-group
Castell et al. [43]–CFS	Analysis: Interventions with more treatment hours were more effective. No difference by intervention length, treatment settings, format, control group, illness duration, diagnostic criteria and study quality.
Malouff et al. [57]–CFS	Analysis: correlations between intervention effectiveness and hours of treatment, number of session, months of follow-up and study quality. Trend of large effect for physical fatigue and small for mental fatigue. No difference between subjective and objective fatigue.
Price et al. [58]—CFS	Analysis: Interventions conducted individually, compared to treatment as usual (versus waiting list) and incorporating increased activity were more effective. No difference by number of sessions.
Picariello et al. [54]–ESKD	Analysis: Interventions with non-fatigued samples at baseline and comparing to active controls did not reduce fatigue, whereas fatigued samples and comparing to non-active controls did. Interventions with facilitators with lower levels of training were more effective. Stress management/relaxation based interventions were more effective than those without. No significant difference by CBT based interventions or not.
Wendebourg et al. [59]–MS	Analysis: CBT based approaches more effective than other techniques (energy conservation, multi-disciplinary self-management, mindfulness). Individual approaches more effective than group settings.
Ulrichsen et al. [52]–Mindfulness	Observation: Intervention studies using fatigue cut-off points and measuring mental fatigue reported increased effects.

### ‘Complementary medicine’ interventions

Fifteen reviews addressed interventions categorised as ‘complementary medicine’ (RA, SLE, IBD, MS, TBI, CFS, Post-stroke, ESKD and PD), although one of these did not include any papers [22](CFS). Further sub-groups were identified;interventions of Eastern origin (e.g. acupuncture, acupuncture based treatment, Chinese herbs, Japanese massage), physiological interventions (e.g. cooling, pulsed electro-magnetic devices, phototherapy), diet and nutritional supplements (e.g. fish oil, acetyl-carnitine). Interventions included under this category in single reviews were reflexology and health tracker information (RA), Qigong (CFS) and Tai Chi and Yoga (mixed) (which other reviews considered under physical activity), continuous positive airway pressure therapy (post-stroke) and occupational therapy (ESKD).

In terms of aim 2, Eastern origin interventions have been investigated in RA (n = 1), SLE (n = 2), CFS (n = 1), Post-stroke (n = 1), ESKD (n = 1) and PD (n = 1). These included herbal medicine (two reviews; RA and CFS), acupuncture, acupuncture related treatments (e.g. minimal needling, electroacupuncture, acupressure, acupoint application) and Japanese massage (one review; PD), all of which were reported as effective in reducing fatigue. Acupuncture/acupuncture based treatment was the most common intervention, applied and reported to be effective across four conditions (all except RA).

Diet manipulation was included in reviews in RA and SLE. In both conditions, diet was reported to be effective in reducing fatigue, specifically a Mediterranean diet [12](RA), a low glycaemic index diet or a low calorie diet [36,48](SLE). Nutritional supplements were reported to not reduce fatigue; omega-3 fish oil (IBD and RA), Vitamin D (SLE). Acetyl-L-carnitine was reported to be more effective for reducing fatigue than amantadine in MS but was not compared to an alternative control group [38], overlapping with Tejani et al. [60] in pharmacological).

Physiological interventions included: electroencephalographic biofeedback, cranial electrotherapy stimulation and bright blue light treatment in TBI [33]; cooling and pulsed electromagnetic therapy in MS [7,30]; and UV phototherapy in SLE [36]. Again, all reviews reported fatigue reduction. These interventions were more novel, identified as effective in individual conditions, addressing aim 3.

### Non-pharmacological study limitations

Reviews highlighted several issues: few studies specifying fatigue as a primary outcome; heterogeneous intervention protocols and study designs (e.g. sample sizes, intervention length, dose, frequency and session duration); single study findings; heterogeneity in control conditions (e.g. waiting list control, active control, treatment as usual); and not considering findings in relation to clinically important differences. For ‘complementary medicine’ interventions in particular, reviews highlighted that most interventions were only investigated by one study, and these studies were often of low quality. This limits the conclusions that can be drawn and highlights the need for caution when interpreting the findings. Potential publication bias was also noted in this group as most interventions, especially the Chinese medicine ones, reported positive findings.

### Fatigue outcome measures in pharmacological and non-pharmacological reviews

Most reviews (n = 44) reported details of fatigue outcome measures, encompassing a large number of diverse measures. These ranged from illness specific measures (e.g. PD fatigue scale, MS Quality of Life Questionnaire, Kidney Disease Questionnaire), single item Likert-type scales, subscales of questionnaires (e.g. POMS (vigor/fatigue), SF-36 (vitality)) and visual analogue scales, to specific fatigue questionnaires such as Chalder Fatigue Scale, Modified Fatigue Impact Scale, Fatigue Severity Scale, Brief Fatigue Inventory and Chronic Illness Therapy Fatigue Domain.

## Discussion

We conducted a comprehensive scoping review mapping fatigue interventions in chronic health conditions, ranging from those where fatigue is a clearly defined key symptom, such as CFS and MS, to less common conditions, such as Sjogren’s syndrome and peripheral neuropathy. In the last five years, there has been an increase in the number of published reviews. However, we note that interventional studies included in reviews often do not specifically target fatigue and include study samples that may not be fatigued at baseline.

Of the pharmacological agents, only anti-TNFs and methylphenidate were identified as beneficial across multiple conditions, in line with aim 2. Anti-TNFs were reported to be effective treatments for fatigue in RA, IBD, Sjogren’s syndrome and sarcoidosis, all conditions classified as autoimmune/inflammatory. Anti-TNF medication acts to suppress the response to the tumour necrosis factor (TNF), for example blocking TNFa (a cytokine which stimulates and regulates inflammation and release of other cytokines as part of the inflammation cascade) to reduce the inflammatory response [61]. Although this medication is targeting inflammatory disease activity as opposed to the fatigue symptom, the cross-condition benefits indicate fatigue mechanisms may be similar, possibly linked to the body’s inflammatory response [62].

In contrast, fluoxetine (an anti-depressant) was reported as ineffective for reducing fatigue in HIV [29], post-stroke [63], mixed palliative care population [24] and CFS [46], suggesting fatigue in these LTCs is not a symptom of depressed mood [11].

Non-pharmacological interventions were particularly heterogeneous, varying in modality, duration and type of intervention provider/facilitator. Even after categorising them into three sub-groups (exercise, psychological/behavioural and ‘complementary medicine’), comparison was hampered by intervention heterogeneity. Broadly speaking, aerobic exercise (as well as general and mixed exercise models that included a substantial aerobic component) and CBT based interventions were the most common and seemed effective in reducing fatigue.

However, none of these were reported to significantly reduce fatigue in three neurological conditions: PD, TBI and post-stroke, indicating mechanisms underlying fatigue in these conditions may be different. Neurological pathology can cause cognitive difficulties, which may account for the ineffectiveness of these interventions in these LTCs [64–66], whereas other non-pharmacological interventions, such as cranial electrotherapy stimulation(which do not rely on patients’ cognitive abilities), were shown to be effective. Programmes such as CBT and graded exercise involve cognitions, planning and goal setting [67]. These may be harder to engage in if attentional or memory deficits are present. This is not to say that cognitive impairment makes exercise and CBT interventions ineffective, as physical activity has been reported to be beneficial for cognition in a meta-analysis in dementia for example [68]. Instead the delivery of these interventions may need structuring in such a way that is accessible for patients.

Review limitations may also account for inconsistencies: i) reviews included studies which had measured fatigue both as a primary (i.e. targeted) outcome or as a secondary outcome; and ii) reviews included a range of measures of fatigue, ranging from fatigue-specific measures to single item statements. As fatigue was often a secondary outcome, interventions may not have been powered to detect changes in fatigue. However, these limitations were not confined to reviews in PD, TBI and post-stroke so do not necessarily account for the inter-condition differences.

### Strengths and limitations

The in-depth search conducted yielded posters, conference abstracts, protocols, unpublished and published articles, and is likely to have captured the relevant research in this area.

However, non-English language papers were not included. Also, of the 64 excluded reviews, 22 investigated CFS. Although fatigue is the condition-defining symptom, most CFS reviews were excluded because they did not specify fatigue as a primary outcome, instead including either a range of undefined outcomes or not explicitly stating outcome of interest (see S6 Table).Cancer-related fatigue was also not included, due to the known association of fatigue with the treatment of the condition. Insights and conclusions from reviews in these conditions will have been missed. One person undertook screening and extraction. A 10% quality check during screening and extraction aimed to minimise the potential of papers or findings being missed, but does not negate the risk completely.

It is not in the scoping review remit to analyse the quality of reviews or individual studies [15], so the review is restrained by reporting; findings for a particular intervention may be based on a single or small number of studies of varying quality, as is the case with interventions from Eastern origin, for example. This highlights the need to bear in mind the aims of the review; to map the current literature and identify patterns, rather than draw definite conclusions about the efficacy of a certain intervention. Nonetheless, the quality of reviews themselves was also variable: inconsistencies in reporting findings from the same studies across reviews arose [34,35,37]; findings were often categorised unclearly and inconsistently; some full texts were not received in response to requests; and some reviews included studies with a non-RCT design, which may result in high risk of bias. Heterogeneity across reviews means it is difficult to assess what the underlying fatigue reduction mechanism may be, and subsequently deduce what components would be needed for an ‘ideal’ fatigue intervention. As per scoping review protocol [15], we did not formally quality assess the reviews to rank/ weight the findings we report so conclusions need to be drawn cautiously.Our findings narratively describe and map the fatigue intervention literature. Thus, only areas of limited research can be identified, which future research could address. Specific recommendations for care pathways cannot be made.

### Implications for future research and practice

Reviewing literature across LTCs highlighted commonalities as well as novel approaches, both of which may be potentially useful for developing interventions. As highlighted by reviews’ recommendations (S1 Table), the methodological quality of the research in this area requires improvement. High quality, guideline adherent RCTs are needed which: recruit participants who are fatigued at baseline; trial interventions that specifically target fatigue as a primary outcome; have a well specified theory/hypothesis regarding the mechanisms through which intervention reduces fatigue; include long term follow up; and report clinical significance.Future reviews should ensure accurate and complete reporting, outline all intervention details and analyse moderators where possible.

In response to aim 2, interventions which may appear most promising for future transdiagnostic research include 1) anti-TNFs and methylphenidate, 2) exercise that is substantially aerobic in nature (either when delivered alone or as part of a general or mixed exercise approach) and graded, 3) psychologically based approaches including CBT, psychoeducation (possibly more so when delivered individually, for increased number of hours and, importantly, when specifically targeting fatigue), 4) diet alteration, 5) acupuncture.

Regarding aim 3, more novel, non-pharmacological strategies which may be worth considering in other LTCs include solution-focussed therapy, expressive writing and guided imagery (if fatigue focussed), and mindfulness.

Although the standard mean differences and their confidence intervals reported in the meta-analyses suggest treatments were generally more efficacious than control (see S2–S5 Tables), effect sizes were only small or moderate in size. Therefore, a multifactorial approach incorporating multiple strategies (for example, a combined exercise-psychological/behavioural-acupuncture-diet programme) may be more effective for reducing fatigue [69] and[70](RA). Interventions need to consider different possible underlying fatigue mechanisms across different LTCs (e.g. neurological versus inflammatory) so a flexible transdiagnostic approach may be needed, taking the pathology and characteristics of the patient populations into account [9]. Importantly, several reviews highlighted that interventions may need to focus specifically on fatigue in order to elicit the most benefit.

### Summary

The aim of a scoping review is to map literature, rather than quality assess or rate evidence, so direct recommendations for practice cannot be made [15].Further research into the effectiveness, safety and cost of interventions would be needed, as would clear identification of mechanisms of action. However, the aims and research questions of this scoping review were addressed. Firstly, the evidence reported by systematic reviews on the topic of fatigue interventions in LTCs was collated and summarised. Secondly, this highlighted the potential transdiagnostic effectiveness of anti-TNFs, methylphenidate, graded aerobic exercise, psychological based approaches, diet and acupuncture. Importantly, the pathology of conditions may determine effectiveness (for example, whether the condition has an autoimmune/inflammatory basis). Lastly, individual condition interventions were identified, of which solution focussed therapy, expressive writing, fatigue focussed guided imagery and mindfulness may be valuable to explore across other conditions. More work is needed to identify transdiagnostic mechanisms of fatigue and to design interventions based on these.

## Appendix A. Database search strategies

### PsycINFO 6

1806- Nov week 1

1. (fatig* adj4 (interven* or improv* or prevent* or program* or therap* or treat* or reduc* or manag*))
2. Exp “Literature Review”/ or review.mp
3. ((systematic or quantitative or scoping or narrative or literature) adj4 (review or overview or synthesis).mp
4. Exp Meta Analysis/
5. (meta-analysis or meta analysis or metaanalysis).mp
6. 2 or 3 or 4 or 5
7. 1 and 6

### EMBASE 6

1974-11/11/2016

1. (fatig* adj4 (interven* or improv* or prevent* or program* or therap* or treat* or reduc* or manag*))
2. “review”/
3. ((systematic or quantitative or scoping or narrative or literature) adj4 (review or overview or synthesis).mp
4. meta analysis/
5. (meta-analysis or meta analysis or metaanalysis).mp
6. 2 or 3 or 4 or 5
7. 1 and 6

### Ovid MEDLINE(R) 6

1946 –Nov week 1 2016

1. (fatig* adj4 (interven* or improv* or prevent* or program* or therap* or treat* or reduc* or manag*))
2. “Review Literature as Topic”/ or review.mp
3. ((systematic or quantitative or scoping or narrative or literature) adj4 (review or overview or synthesis).mp
4. Exp Meta Analysis/
5. (meta-analysis or meta analysis or metaanalysis).mp
6. 2 or 3 or 4 or 5
7. 1 and 6

### Ovid MEDLINE(R) In process and other non-indexed citations 6

Until Nov 9^th^ 2016

1. (fatig* adj4 (interven* or improv* or prevent* or program* or therap* or treat* or reduc* or manag*))
2. Review.mp or exp “Review”/
3. ((systematic or quantitative or scoping or narrative or literature) adj4 (review or overview or synthesis).mp
4. Exp Meta Analysis/
5. (meta-analysis or meta analysis or metaanalysis).mp
6. 2 or 3 or 4 or 5
7. 1 and 6

### Cochrane

(-> 18/11/2016)

N = 223 (reviews and protocols)

N = 117 (‘other’ reviews)

‘fatigue’

### CINAHL

1900–2016 present (14/11/2016)

N = 436

TX (fatig* N4 (interven* or improv* or prevent* or program* or therap* or treat* or reduc* or manag*)) AND TX ((systematic or quantitative or scoping or narrative or literature) N4 (review or overview or synthesis) or meta analysis)

### Web of science

All years

N = 485

**TOPIC:** (fatig* near/4 (interven* or improv* or prevent* or program* or therap* or treat* or reduc* or manag*)) *AND***TOPIC:** (meta analysis OR ((systematic or quantitative or scoping or narrative or literature) NEAR/4 (review or overview or synthesis)))

### LILACS

N = 30

N = 3 in English (none of those relevant). 1 is potentially relevant but article in Spanish:

Efectividad del ejercicio físico en la fatiga de pacientes con cáncer durante el tratamiento activo: revisión sistemática y metaanálisis / Effectiveness of physical exercise on fatigue in cancer patients during active treatment: a systematic review and meta-analysis / Eficácia do exercício físico na fadiga dos pacientes com câncer durante o tratamento ativo: revisão sistemática e meta-análise

Meneses-Echávez, Jose Francisco; González-Jiménez, Emilio; Correa-Bautista, Jorge Enrique; Valle, Jacqueline Schmidt-Río; Ramírez-Vélez, Robinson.

Cad Saude Publica; 31(4): 667–681, 04/2015. tab, graf

Article in Spanish | LILACS | ID: lil-744849

(fatigue) AND (intervention OR improve OR prevent OR program OR therap OR treat OR reduc OR manag) AND (review)

### PEDro

fatig* interven* (Method selected from drop down list = systematic review)

N = 191

### OpenGrey 12/12/16

“Fatigue [intervention/treatment/management/prevention/therapy/program/improve] Review”

N = 9. None relevant.

### Google

Consultation suggested to check:

- Ankylosing spondylitis– 1 –for full text review
- SLE– 1- (already captured in database search)
- Polymyositis– 0
- Vasculitis– 0

Review Fatigue [Intervention/Treatment] ([condition])

New/previously not found: n = 3 for full text screening.

## Supporting information

S1 Text References of studies included in scoping review. (DOCX)

S2 Text List of protocols identified. (DOCX)

S1 Table Review characteristics. (DOCX)

S2 Table Reviews including pharmacological interventions for fatigue treatment. (DOCX)

S3 Table Reviews including exercise interventions for fatigue treatment. (DOCX)

S4 Table Reviews including psychological/behavioural interventions for fatigue treatment. (DOCX)

S5 Table Reviews including complementary medicine non-pharmacological interventions for fatigue treatment. (DOCX)

S6 Table Excluded reviews. (DOCX)

## Abbreviations

LTCs: Long term conditions
HIV: Human immunodeficiency virus
RA: Rheumatoid arthritis
MS: Multiple sclerosis
JBI: Joanna Briggs Institute
RCTs: Randomised controlled trials
CFS: Chronic fatigue syndrome
DHEA: Dehydroepiandrosterone
TBI: Traumatic brain injury
IBD: Inflammatory bowel disease
ESKD: End stage kidney disease
SLE: Systemic lupus erythematosus
TNF: Tumour necrosis factor
PD: Parkinson’s disease
GET: Graded exercise therapy
CBT: Cognitive behavioural therapy
