# Supplementary material for: Fatigue interventions in long term, physical health conditions: A scoping review of systematic reviews
Source: PLoS One. 2018 Oct 12;13(10):e0203367. doi: 10.1371/journal.pone.0203367 (PMC6193578; doi:10.1371/journal.pone.0203367)
Supplement: S1 Text — (DOCX) [file pone.0203367.s001.docx]

**S1 Text. References of studies included in scoping review**

The following references were included in the review but not referenced directly in the

manuscript:

Asano M, Finlayson Ml. Meta-analysis of three different types of fatigue management interventions for people with multiple sclerosis: exercise, education, and medication (Provisional abstract). Database of Abstracts of Reviews of Effects. 2014; 2: 798285. http://onlinelibrary.wiley.com/o/cochrane/cldare/articles/DARE-12014040429/frame.html

Astroth KS, Russell CL, Welch JL. Non-pharmaceutical fatigue interventions in adults receiving hemodialysis: A systematic review. Nephrology Nursing Journal. 2013; 40(5): 407-427.

Kohli IS, Kataria A, Singla S, Kaushik P, Jindal R, Aggarwal A. Effect of cognitive behavioural therapy in multiple sclerosis fatigue: A systematic review of randomised controlled trials. Value in Health. 2012; 15(7): A545-A546.

Pilutti LA, Greenlee TA, Motl RW, Nickrent MS, Petruzzello SJ. Effects of exercise training on fatigue in multiple sclerosis: A meta-analysis. Psychosomatic Medicine. 2013; 75(6): 575-580.

Pucci E, Branas P, D'Amico R, Giuliani G, Solari A, Taus C. Amantadine for fatigue in multiple sclerosis. Cochrane Database of Systematic Reviews 1. 2007; CD002818: DOI: 10.1002/14651858.CD002818.pub2.

Seneviratne AC, Bhalara S. What is the most beneficial treatment of fatigue in primary Sjogren's syndrome? a systematic review. Rheumatology (United Kingdom). 2014; 53: i135.

Wang YY, Li XX, Liu JP, Luo H, Ma LX, Alraek T. Traditional Chinese medicine for chronic fatigue syndrome: A systematic review of randomized clinical trials. Complementary Therapies in Medicine. 2014; 22(4): 826-833.

White CM, Van Doorn PA, Garssen MPJ, Stockley RC. Interventions for fatigue in peripheral neuropathy. Cochrane Database of Systematic Reviews 4. 2014; CD008146: DOI: 10.1002/14651858.CD008146.pub2.

The following reviews were referenced in the manuscript:

Adams D, Wu T, Yang X, Tai S, Vohra S. Traditional Chinese medicinal herbs for the treatment of idiopathic chronic fatigue and chronic fatigue syndrome. Cochrane Database of Systematic Reviews 4. 2009; (CD006348). DOI: 0.1002/14651858.CD006348.pub2.

Almeida C, Choy EH, Hewlett S, Kirwan JR, Cramp F, Chalder T, Pollock J, Christensen R. Biologic interventions for fatigue in rheumatoid arthritis. The Cochrane database of systematic reviews 4. 2016; CD008334. DOI: 10.1002/14651858.CD008334.pub2.

Andreasen AK, Stenager E, Dalgas U. The effect of exercise therapy on fatigue in multiple sclerosis. Multiple Sclerosis Journal. 2011; 17(9): 1041-1054.

Artom M, Czuber-Dochan W, Sturt J, Norton C. Targets for Health Interventions for Inflammatory Bowel Disease-fatigue. Journal of Crohn's & Colitis. 2016; 10(7): 860-869. doi:10.1093/ecco-jcc/jjw029

Asano M, Berg E, Johnson K, Turpin M, Finlayson M. A scoping review of rehabilitation interventions that reduce fatigue among adults with multiple sclerosis. Disability and Rehabilitation: An International, Multidisciplinary Journal. 2015; 37(9): 729-738.

Atkins C, Wilson AM. Managing fatigue in sarcoidosis – a systematic review of the evidence. Chronic Respiratory Disease. 2016; 1-13: doi: 10.1177/1479972316661926

Blikman LJ, Huisstede BM, Kooijmans H, Stam HJ, Bussmann JB, Meeteren J. Effectiveness of energy conservation treatment in reducing fatigue in multiple sclerosis: a systematic review and meta-analysis (Structured abstract). Archives of Physical Medicine and Rehabilitation. 2013; 94(7): 1360-1376.

Brañas P, Jordan R, Fry-Smith A, Burls A, Hyde C. Treatments for fatigue in multiple sclerosis: a rapid and systematic review. Health Technology Assessment. 2000; 4(37): 1-61.

Brown JN, Howard CA, Kemp DW. Modafinil for the treatment of multiple sclerosis-related fatigue. Annals of Pharmacotherapy. 2010; 44(6): 1098-1103.

Bruno AE, Sethares KA. Fatigue in Parkinson disease: An integrative review. Journal of Neuroscience Nursing. 2015; 47(3): 146-153. DOI: 10.1097/JNN.0000000000000131

Cantor JB, Ashman T, Bushnik T, Xinsheng C, Farrell-Carnahan L, Gumber S, Hart T, Rosenthal J, Dijkers MP. Systematic Review of Interventions for Fatigue After Traumatic Brain Injury: A NIDRR Traumatic Brain Injury Model Systems Study. Journal of Head Trauma Rehabilitation. 2014; 29(6): 490-497. doi: 10.1097/HTR.0000000000000102

Castell BD, Kazantzis N, Moss-Morris RE. Cognitive behavioral therapy and graded exercise for chronic fatigue syndrome: A meta-analysis. Clinical Psychology: Science and Practice. 2011; 18(4): 311-324.

Chauffier K, Salliot C, Berenbaum F, Sellam J. Effect of biotherapies on fatigue in rheumatoid arthritis: A systematic review of the literature and meta-analysis. Rheumatology. 2012; 51(1): 60-68.

Cleanthous S, Tyagi M, Isenberg D, Newman S. What do we know about self-reported fatigue in systemic lupus erythematosus? Lupus. 2012; 21: 465-476.

Cleare AJ, Reid S, Chalder T, Hotopf M, Wessely S. Chronic fatigue syndrome. Clinical Evidence. 2015; 9(1101): 1-40

Cramp F, Hewlett S, Almeida C, Kirwan JR, Choy EHS, Chalder T, Pollock J, Christensen R. Non-pharmacological interventions for fatigue in rheumatoid arthritis. Cochrane Database of Systematic Reviews 8; 2013: doi: 10.1002/14651858.CD008322.pub2

del Pino-Sedeno, T., Trujillo-Martin, M. M., Ruiz-Irastorza, G., Cuellar-Pompa, L., de Pascual-Medina, A. M., Serrano-Aguilar, P., & The Spanish Systemic Lupus Erythematosus, C. P. G. Development Group. Effectiveness of nonpharmacologic interventions for decreasing fatigue in adults with systemic lupus erythematosus: a systematic review. Arthritis Care & Research. 2016; 68(1): 141-148. DOI 10.1002/acr.22675

Elbers RG, van Verhoef J, Wegen Erwin Eh B, Henk W, Kwakkel G. Interventions for fatigue in Parkinson's disease. Cochrane Database of Systematic Reviews 10. 2015; CD010925. DOI:10.1002/14651858.CD010925.pub2

Franssen M, Winward C, Collett J, Wade D, Dawes H. Interventions for fatigue in Parkinson's disease: A systematic review and meta-analysis. Movement Disorders. 2014; 29(13): 1675-1678. DOI: 10.1002/mds.26030

Godhrawala H. Efficacy of resistance training on fatigue and quality of life in multiple sclerosis patients-a systematic review. Physiotherapy (United Kingdom). 2007; 97: eS413.

Heine M, van de Port I, Rietberg MB, van Wegen EEH, Kwakkel G. Exercise therapy for fatigue in multiple sclerosis. Cochrane Database of Systematic Reviews 9. 2015; doi: 10.1002/14651858.CD009956.pub2

Jong E, Oudhoff LA, Epskamp C, Wagener MN, van Duijn M, Fischer S, van Gorp EC. Predictors and treatment strategies of HIV-related fatigue in the combined antiretroviral therapy era. AIDS. 2010; 24(10): 1387-1405.

Khan F, Amatya B, Galea M. Management of fatigue in persons with multiple sclerosis. Frontiers in Neurology. 2014; 5(177): doi: 10.3389/fneur.2014.00177

Larun L, Brurberg KG, Odgaard-Jensen J, Price JR. Exercise therapy for chronic fatigue syndrome (Cochrane review). Cochrane Database of Systematic Reviews 6. 2016; CD003200. DOI: 10.1002/14651858.CD003200.pub5.

Latimer-Cheung AE, Pilutti LA, Hicks AL, Martin Ginis KA, Fenuta AM, MacKibbon A, Motl RW. Effects of exercise training on fitness, mobility, fatigue, and health-related quality of life among adults with multiple sclerosis: a systematic review to inform guidelines development. Acrhives of Physical Medicine and Rehabilition. 2013; 94: 1800-1828.

Lee D, Newell R, Ziegler L, Topping A. Treatment of fatigue in multiple sclerosis: A systematic review of the literature. International Journal of Nursing Practice. 2008; 14(2): 81-93.

Malouff JA, Thorsteinsson EB, Rooke SE, Bhullar N, Schutte NS. Efficacy of cognitive behavioral therapy for chronic fatigue syndrome: A meta-analysis. Clinical Psychology Review. 2008; 28(5): 736-745. doi: 10.1016/j.cpr.2007.10.004

Marques MM, de Gucht V, Gouveia MJ, Leal I, Maes S. Differential effects of behavioral interventions with a graded physical activity component in patients suffering from chronic fatigue (syndrome): an updated systematic review and meta-analysis. Clinical Psychology Review. 2015; 40: 123-137.

Menzies V, Jallo, N. Guided imagery as a treatment option for fatigue: a literature review. Journal of holistic nursing : official journal of the American Holistic Nurses' Association. 2011; 29(4): 279-286.

Mücke M, Cuhls H, Peuckmann-Post V, Minton O, Stone P, Radbruch L. Pharmacological treatments for fatigue associated with palliative care. The Cochrane database of systematic reviews 5. 2015; CD006788. DOI: 10.1002/14651858.CD006788.pub3.

Neill J, Belan I, Ried K. Effectiveness of non-pharmacological interventions for fatigue in adults with multiple sclerosis, rheumatoid arthritis, or systemic lupus erythematosus: A systematic review. Journal of Advanced Nursing. 2006; 56(6): 617-635.

Picariello F, Hudson JL, Moss-Morris R, Macdougall IC, Chilcot J. Examining the efficacy of social-psychological interventions for the management of fatigue in end-stage kidney disease (ESKD): a systematic review with meta-analysis. Health Psychology Review [online]. 2017: DOI: 10.1080/17437199.2017.1298045

Price JR, Mitchell E, Tidy E, Hunot V. Cognitive behaviour therapy for chronic fatigue syndrome in adults (Cochrane review) [with consumer summary]. Cochrane Database of Systematic Reviews 3. 2008; CD001027: DOI: 10.1002/14651858.CD001027.pub2.

Puetz TW, Beasman KM, O'Connor PJ. The effect of cardiac rehabilitation exercise programs on feelings of energy and fatigue: a meta-analysis of research from 1945 to 2005. European Journal of Cardiovascular Prevention and Rehabilitation. 2006; 13(6): 886-893.

Russell D, Alvarez Gallardo IC, Hughes CM, Davison GW, Corrales BS, McVeigh JG. The effectiveness of exercise in the management of fatigue and sleep dysfunction in fibromyalgia syndrome: A systematic review. Rheumatology (United Kingdom). 2014; 53: i146.

Sheng P, Hou LJ, Wang X, Wang XW, Huang CG, Yu MK, Han X, Dong Y. Efficacy of Modafinil on Fatigue and Excessive Daytime Sleepiness Associated with Neurological Disorders: A Systematic Review and Meta-Analysis. Plos One. 2013; 8(12); doi: 10.1371/journal.pone.0081802

Smith C, Hale L. The effects of non-pharmacological interventions on fatigue in four chronic illness conditions: a critical review. Physical Therapy Reviews. 2007; 12(4): 324-334.

Tejani AM, Wasdell M, Spiwak R, Rowell G, Nathwani S. Carnitine for fatigue in multiple sclerosis. Cochrane database of systematic reviews 5. 2012; CD007280: DOI: 10.1002/14651858.CD007280.pub3.

Ulrichsen KM, Kaufmann T, Dorum ES, Kolskar KK, Richard G, Alnaes D, Arneberg TJ, Westlye LT, Nordvik, Jan E. Clinical utility of mindfulness training in the treatment of fatigue after stroke, traumatic brain injury and multiple sclerosis: A systematic literature review and meta-analysis. Frontiers in Psychology. 2016; 7: doi: 10.3389/fpsyg.2016.00912.

van den Akker LE, Beckerman H, Collette EH, Eijssen IC, Dekker J, de Groot V. Effectiveness of cognitive behavioral therapy for the treatment of fatigue in patients with multiple sclerosis: A systematic review and meta-analysis. Journal of Psychosomatic Research. 2016; 90: 33-42.

Wendebourg MJ, Heesen C, Finlayson M, Meyer B, Pottgen J, Kopke S. Patient education for people with multiple sclerosis associated fatigue: A systematic review. PLOS One. 2017; 12(3): e0173025. doi:10.1371/journal.pone.0173025

Yuen HK, Cunningham MA. Optimal management of fatigue in patients with systemic lupus erythematosus: A systematic review. Therapeutics and Clinical Risk Management. 2014; 10: 775-786.

Wu S, Kutlubaev MA, Chun HY, Cowey E, Pollock A, Macleod MR, Dennis M, Keane E, Sharpe M, Mead GE. Interventions for post-stroke fatigue: A systematic review and meta-analysis. International Journal of Stroke. 2015; 10, CD007030: DOI: 10.1002/14651858.CD007030.pub3.
