# Supplementary material for: Fatigue interventions in long term, physical health conditions: A scoping review of systematic reviews
Source: PLoS One. 2018 Oct 12;13(10):e0203367. doi: 10.1371/journal.pone.0203367 (PMC6193578; doi:10.1371/journal.pone.0203367)
Supplement: S2 Text — (DOCX) [file pone.0203367.s002.docx]

**S2 Text. List of protocols identified**

**Eight protocols were identified. Authors indicated four reviews were on-going/in preparation:**

Almeida C, Chalder T, Cramp F, Choy EHS, Kirwan JR, Hewlett S, Pollock J, Christensen R. Non-biologic pharmaceutical interventions for fatigue in rheumatoid arthritis (Protocol). Cochrane Database of Systematic Reviews 10. 2010; CD008795: doi:10.1002/14651858.CD008795

Farrell D, Savage E, Norton C, Jelsness-Jørgensen LP, Czuber-Dochan W, Artom M. Interventions for fatigue in inflammatory bowel disease (Protocol). Cochrane Database of Systematic Reviews 12. 2015; CD012005: doi:10.1002/14651858.CD012005

Moss-Morris R, Mercer T, White C, Thomas S, Van den Linden M, Harrison A, Safari R, Norton S. Which exercise or behavioural fatigue interventions are effective for people with multiple sclerosis (MS)? A systematic review with detailed intervention breakdown and meta-analysis. PROSPERO. 2016a; CRD42016033763.

Moss-Morris R, Mercer T, White C, Van den Linden M, Thomas S, Harrison A, Safari R, Norton S. Efficacy of targeted versus non-targeted exercise and behavioural interventions on fatigue in multiple scleorsis: systematic review and meta-analaysis. PROSPERO. 2016b; CRD42016036671.

**Author replies were not received regarding the remaining four:**

Caceres C, Ferguson L, Fernandez R. The effectiveness of the non-pharmacological management of fatigue in adults with multiple sclerosis (Review Protocol). JBI Library of Systematic Reviews. 2008; 6(4): 21.

Hicks E, Senior HE, Purdy S, Barker-Collo S, Larkins B. Interventions for fatigue management after traumatic brain injury (Protocol). Cochrane Database of Systematic Reviews 2. 2007: CD006448. doi:10.1002/14651858.CD006448

Tan H, Wu X, Wei D, Luo T, Qu Y, Wen M, Lan Y. Systematic review of acupuncture for chronic fatigue syndrome. PROSPERO. 2015: CRD42015015229.

Young CA, Gibbons C, Pagnini F, Friede T. Treatment for fatigue in amyotrophic lateral sclerosis/motor neuron disease (ALS/MND) (Protocol). Cochrane Database of Systematic Reviews 3. 2014: CD011005. doi:10.1002/14651858.CD011005
