# Supplementary material for: Fatigue interventions in long term, physical health conditions: A scoping review of systematic reviews
Source: PLoS One. 2018 Oct 12;13(10):e0203367. doi: 10.1371/journal.pone.0203367 (PMC6193578; doi:10.1371/journal.pone.0203367)
Supplement: S1 Table — (DOCX) [file pone.0203367.s003.docx]

| **S1 Table. Characteristics of included reviews.** | | | | | | |
| --- | --- | --- | --- | --- | --- | --- |
| **Reference** | **Health condition** | **Type of review – including method of synthesis** | **Date assessed up to - last search** | **Intervention focus**  **(Pharmacological, Non-pharmacological, Mixed)** | **Number and type of fatigue intervention studies included in the review** | **Summary of review recommendations for future research** |
| Adams et al. 2010 | Idiopathic fatigue and chronic fatigue syndrome | Systematic - narrative | August 2008 (Assessed as up to date: January 2009) | Non-pharmacological | 0 | Improvement in methodology and reporting of future trials.  Caution of publication bias. |
| Almeida et al. 2016 | RA | Systematic with meta-analysis | April 2014 | Pharmacological | 32  All randomised controlled trials (RCTs) | Need to determine mechanisms of action.  Assess long term improvement. |
| Andreasen et al. 2011 | MS | Systematic - narrative | July 2010 | Non-pharmacological | 21  10 RCTs, 7 non-controlled, remaining were controlled, cross over or single subject trials. | Evaluate fatigue as a primary outcome. Use fatigued populations. RCTs with fatigue as primary endpoint. Fatigue scales should be multi-dimensional and have well established cut offs. |
| Artom et al. 2016 | IBD | Systematic overview - narrative | Not clear | Mixed | 8  7 RCTs, 1 open-label pilot | Develop integrated working models addressing multiple modifiable aspects and interactions between them. |
| Asano et al. 2015 | MS | Scoping review | July 2013 | Non-pharmacological | 38  20 of these were RCTs | Adequately powered future trials. Investigate combining exercise and behaviour interventions. |
| Asano & Finlayson 2014 | MS | Systematic with meta-analysis | August 2013 | Mixed | 25  All RCTs | Investigate different patient subgroups and exercise formats to optimise effectiveness. |
| Astroth et al. 2016 | ESKD (receiving haemodialysis) | Systematic - narrative | November 2011 | Non-pharmacological | 25  11 RCTs, 14 quasi-experimental | Fully powered RCTs, consistency in reporting measures, theory based interventions. |
| Atkins & Wilson 2016 | Sarcoidosis | Systematic – narrative | November 2015 | Mixed | 8  3 RCTs, 3 case series, 2 retrospec. (case review and observational) | Trials with sufficient sample size and duration |
| Blikman et al. 2013 | MS | Systematic with meta-analysis | May 2012 | Non-pharmacological | 6  4 RCTs, 2 controlled clinical trials (CCTs). | High quality RCTs that also study long term effects. |
| Branas et al. 2000  a)  b) | MS | Scoping review  Systematic - narrative | June 1999  December 1999 | Mixed  Pharmacological | 15; 13 RCTs, 2 case series  6, all RCTs | Further rigorous research needed on the effectiveness of the most usual initial clinical management, behavioural advice for this significant symptom.  Investigate long term benefits and side effects. |
| Brown et al. 2010 | MS | Systematic - narrative | January 2010 | Pharmacological | 6  3 open labelled trials, 1 single blind, 2 RCTs | Larger RCTs to investigate appropriate dose, long term effects and adverse effects. Comparator studies. |
| Bruno & Sethares. 2015 | Parkinson’s Disease | Systematic – narrative | 2013 | Mixed | 7  5 randomised or control studies, 1 case series, 1 cross-section. | Clear definition of fatigue. Larger studies with representative samples. Interventions with statistically and clinically significant effect. |
| Cantor et al. 2014 | TBI | Systematic - narrative | January 2014 | Mixed | 19  9 RCTs, remaining include pre-post study, case series and open label pilot | Operational definition of PTBI fatigue. Studies designed with fatigue as primary outcome. Systematic measurements of fatigue. Constrain around severity and duration to reduce variability around intervention delivery. |
| Castell et al. 2011 | CFS | Systematic with meta-analysis | April 2011 | Non-pharmacological | 20  All RCTs | Compare efficacy and cost benefit. Effect of therapy quality/experience and therapist influences. Compare treatment settings and therapy types. Mediators and mechanisms need to be investigated further. |
| Chauffier et al. 2012 | RA | Systematic with meta-analysis | October 2009 | Pharmacological | 10  All RCTs | More RCTs. Systematically include fatigue assessment as outcome. Multi-module approach should be evaluated. |
| Cleanthous et al. 2012 | SLE | Systematic overview – narrative | 2010 | Non-pharmacological | 10  Not given | Longer follow up studies. Consensus on fatigue measurement – instruments need to cover all relevant dimensions. Prospective design. |
| Cleare et al. 2015 | CFS | Systematic - narrative | November 2013 | Mixed | 18  RCTs or systematic reviews | None given – Clinical review. |
| Cramp et al. 2013 | RA | Systematic with meta-analysis | October 2102 | Non-pharmacological | 24  All RCTs | High quality RCTs, with cost-effectiveness analysis. Report in line with CONSORT statement, including adverse events and side effects. Consideration of potential causal pathways and mechanisms of fatigue. Outcome measures need to be standardised. Focus specifically on fatigue. |
| Del Pino-Sedeno et al. 2016 | SLE | Systematic - narrative | June 2014 | Non-pharmacological | 12  7 randomised, 1 non-randomised, 4 prospective observational. | More studies needed to verify promising results. |
| Elbers et al. 2015 | Parkinson’s Disease | Systematic with meta-analysis | April 2015 | Mixed | 11  All RCTs | Improved quality of evidence. Focus on interventions that address maladaptive behavioural and cognitive aspects. Consider characteristics to identify responders and non-responders. |
| Franssen et al. 2014 | Parkinson’s Disease | Systematic with meta-analysis | November 2013 | Mixed | 14  All RCTs | Improvement in fatigue definition and measurement. |
| Godhrawala et al. 2010 | MS | Abstract.  Systematic - narrative | November 2008 | Non-pharmacological | 8  Not given | Standardised, well validated MS specific fatigue measures. Improve methodological and scientific rigour. Account for types of MS, dose and disparity between exercise and control groups. |
| Heine et al. 2015 | MS | Systematic with meta-analysis | October 2014 | Non-pharmacological | 45  All RCTs | High quality, randomised design trials. Specifically targeted to fatigued people with MS. Follow CONSORT guidelines for reporting. Investigate long term outcomes. |
| Jong et al. 2010 | HIV | Systematic - narrative | August 2008 | Mixed | 12  6 RCTs, 6 controlled. | Fatigue measurement. Comparison of medication and behavioural interventions. |
| Khan et al. 2014 | MS | Systematic overview - narrative | June 2014 | Mixed | 27  12 systematic reviews, 12 RCTs, 2 CCT, 1 comparative study | Identify who benefits. Larger studies to confirm effectiveness. Investigate feasibility and long term use and long term follow up. More high quality RCTs. |
| Kohli et al. 2012 | MS | Poster  Systematic with meta-analysis | June 2012 | Non-pharmacological | 4  All RCTs | Large RCTs with long follow up. Investigate appropriate time and most effective modes of delivery, and whether targeting any co-existing condition improves fatigue. |
| Larun et al. 2016 | CFS | Systematic with meta-analysis | May 2014 | Non-pharmacological | 8  All RCTs | Randomised trials with low risk of bias. Investigate components of beneficial exercise intervention. Report contextual characteristics. |
| Latimer-Cheung et al. 2013 | MS | Systematic - narrative | March 2011 | Non-pharmacological | 30  15 RCTs, 15 non-RCTs | Strategic selection of meaningful measures. Investigate populations with more severe disability – development of adapted exercise strategy for this group. |
| Lee et al. 2008 | MS | Systematic - narrative | not given | Mixed | 15  (RCTs, open label and cross-over trials included) | Address methodological difficulties, e.g. waiting list controls, crude fatigue ratings. |
| Malouff et al. 2008 | CFS | Systematic with meta-analysis | June 2007 | Non-pharmacological | 13  Randomised assignment. | Investigate components’ effect on outcome – e.g. hours, gradual increase. Investigate which methods reduce drop out. How to increase % of patients who move and stay in normal fatigue range. Important differences between individuals who meet different criteria? Provide detailed information: treatment details, effect size, sample sizes |
| Marques et al. 2015 | CFS | Systematic with meta-analysis | 2013 | Non-pharmacological | 14  All controlled studies. | Explore potential moderators. Present sufficient detail, effect sizes and raw data. |
| Menzies & Jallo, 2013 | Mixed  (asthma, bronchitis and emphysema, HIV, MS, heart failure, cancer) | Scoping review | June 2008 | Non-pharmacological  (Specifically guided imagery) | 8  3 RCTs, remaining were repeated measures, pre-post, or controlled. | Standardising interventions according to duration and exposure. Targeted fatigue imagery. Studies adequately powered to detect changes. |
| Mücke et al. 2016 | Palliative care (MS, HIV/AIDS, cancer, post-polio, Parkinson’s, ESKD, ALS, COPD) | Systematic with meta-analysis | April 2014 | Pharmacological | 45  All RCTs | Further research to replicate/investigate clinical efficacy of medications identified. Higher number of participants in trials. Agree on use of a particular measure. |
| Neill et al. 2006 | MS, RA, SLE | Systematic - narrative | June 2006 | Non-pharmacological | 36 papers of 33 studies  19 quasi-experimental, 14 RCTs | Improvement in methodological quality, and reporting that conforms to guidelines (e.g. CONSORT). Agreement on appropriate instruments. Comparable studies to enable meta-analysis. |
| Picariello et al. 2017 | ESKD | Systematic with meta-analysis | December 2015 | Non-pharmacological | 16  All RCTs | Interventions need to focus on fatigue and be conducted with fatigued samples. Use of instrument validated in condition. Report trials in line with CONSORT. Need a clear framework on what beliefs and behaviours should be targeted in treatment. |
| Pilutti et al. 2013 | MS | Systematic with meta-analysis | October 2012 | Non-pharmacological | 17  All RCTs | Investigate which mode of exercise is most effective. |
| Price et al. 2008 | CFS | Systematic with meta-analysis | March 2008 | Non-pharmacological | 15  All RCTs | Larger, appropriately powered samples. Use of treatment as usual as control rather than waiting list. Systematic assessment of therapy to manuals/protocols. Trials of CBT+ combination interventions. Consider settings – CBT effective for severely disabled? |
| Pucci et al. 2009 | MS | Systematic - narrative | July 2006 | Pharmacological | 5  3 RCTs, 1 randomised parallel trial, 1 randomised crossover trial. | Adequate sample size. Parallel arm RCTs, clinically relevant, reliable, responsive outcome measures. Definition needs to be better specified. Follow specific guidelines for methodology and reporting. |
| Puetz et al. 2006 | Coronary heart disease | Systematic with meta-analysis | May 2005 | Non-pharmacological | 36  9 RCTs, 6 randomised, 3 non-RCTs, 18 pretest -posttest. | More data from exercise alone interventions needed for comparison – moderator variables. Investigate which component accounts for most of the improvement in fatigue. Further research to examine association between anxiety, depression and fatigue and their relative importance in overall psychological changes. Which exercise characteristics maximise the impact of interventions. |
| Russel et al. 2014 | Fibromyalgia | Abstract  Systematic – narrative | Not given | Non-pharmacological | 35  Not given | None given. |
| Seneviratne & Bhalara, 2014 | Primary Sjogren’s Syndrome | Abstract  Systematic – narrative | 2013 | Pharmacological | 15  8 RCTs, 7 prospective | Larger trials. Aimed at multi-dimensional approach, including psychological input. |
| Sheng et al. 2013 | Neurological disorders (Parkinson’s, MS, TBI, post-polio syndrome) | Systematic with meta-analysis | December 2012 | Pharmacological | 10  All RCTs | Assess safety of modafinil long term administration. Large RCTs – exclude sleep disorders as confounders. |
| Smith & Hale 2007 | Mixed  (MS, Parkinson’s, HIV/AIDS, cancer) | Systematic overview - narrative | 2006 | Non-pharmacological | 27  Various designs including RCTs, pre-post-group, pilot studies. | Consistency of fatigue measurement scales. |
| Tejani et al. 2012 | MS | Systematic - narrative | September 2011 | Pharmacological | 1  RCT | Need to be based on good design principles. Minimal clinically important differences need to be identified for common fatigue measures. |
| Ulrichsen et al. 2016 | Stroke, TBI, MS | Systematic with meta-analysis | May 2016 | Non-pharmacological  (Specifically mindfulness) | 4  Randomised/ quasi-randomised control trials to be included. | Investigate inter-condition differences e.g. age, medication, lifestyle. |
| van den Akker et al. 2016 | MS | Systematic with meta-analysis | February 2016 | Non-pharmacological | 6  All RCTs | Investigate how effects can be preserved to maintain benefits long term. Investigate whether deliver mode affects treatment (moderators). |
| Wang et al. 2014 | CFS | Systematic with meta-analysis | September 2013 | Non-pharmacological | 23  All RCTs | Clinical trials should prospectively register in international trial registery. Appropriate control group and blinding. Use outcomes which are clearly defined, clinically relevant and validated. Methodological clarity. Large, well designed, high quality studies to confirm findings. |
| Wendebourg et al. 2017 | MS | Systematic with meta-analysis | January 2016 | Non-pharmacological | 10  All RCTs | Longer term follow up. Investigate how difference elements can be combined to optimize effects of complex interventions. |
| White et al. 2014 | Peripheral neuropathy (Charcot-Marie Tooth disease, Guillian-Barre Syndrome) | Systematic with meta-analysis | November 2013 | Pharmacological  (any searched but only pharma included) | 3  All RCTs | Larger studies might improve power. Adequate sample size, appropriate randomised controlled designs and clinically relevant, standardised and responsive measures of fatigue as an outcome. |
| Wu et al. 2015 | Post-stroke | Systematic with meta-analysis | May 2014 | Mixed | 12  All RCTs | Further explore those pharmacological interventions which showed efficacy. Investigate efficacy of combined interventions. Future RCTs need more robust design. |
| Yuen & Cunningham 2014 | SLE | Systematic - narrative | May 2014 | Mixed | 26  18 RCTs, remaining included pretest-posttest, random. comparative trial, open label observ. | Large scale RCTs with long term follow up to further investigate interventions identified as potentially effective. |
