# Supplementary material for: Fatigue interventions in long term, physical health conditions: A scoping review of systematic reviews
Source: PLoS One. 2018 Oct 12;13(10):e0203367. doi: 10.1371/journal.pone.0203367 (PMC6193578; doi:10.1371/journal.pone.0203367)
Supplement: S2 Table — (DOCX) [file pone.0203367.s004.docx]

**S2 Table. Reviews including pharmacological interventions for fatigue treatment.**

| **Author/date/type of review/remarks** | **Focus of review** | **Pharmacological agents** | **Summary of findings** | **Implications** |
| --- | --- | --- | --- | --- |
| Rheumatoid Arthritis | | | | |
| Almeida et al., 2016  Systematic review – meta-analysis | Biologic interventions | Anti-TNF biologic agents (adalimumab, certolizumab, etanercept, golimumab, infliximab)  Non-anti TNF biologic agents (abatacept, canakinumab, rituximab, tocilizumab, anti-interferon gamma monoclonal antibody) | Overall, biologics significantly reduced fatigue (Standard mean difference (SMD) = −0.43; 95% CI −0.38, −0.49).  **Subgroup**  Similar reduction in fatigue for Anti-TNFs: SMD = −0.42 (95% CI −0.35, −0.49), and non-anti-TNFs: SMD= −0.46 (95% CI −0.39, −0.53). | Anti-TNFs and non-anti-TNFs to the same extent may be an effective way to reduce fatigue. |
| Chauffier et al. 2012  Systematic review – meta-analysis  (study overlap – all studies included were included in Almeida et al., 2016) | Biotherapies | Anti-TNFs (adalimumab, golimumab, certolizumab)  Non-anti-TNFs (tocilizumab, rituximab, abatacept) | Overall, biotherapies significantly reduced fatigue (SMD=0.45; 95% CI 0.31, 0.58).  **Subgroup**  Non-anti TNFs (SMD=0.57; 95% CI 0.39, 0.75) more effective than anti-TNFs  (SMD=0.36; 95% CI 0.21, 0.51). | Anti-TNFs and non-anti-TNFs may be effective in fatigue reduction, non-anti-TNFs possibly more effective. |
| Inflammatory Bowel Disease | | | | |
| Artom et al., 2016  Systematic overview, including treatment of fatigue | Mixed – any interventions included. | Thiamine, anti-TNFs (infliximab, adalimumab) | Thiamine and Anti-TNFs significantly reduced fatigue. | Thiamine and Anti-TNFs may be effective in reducing fatigue. |
| Sarcoidosis | | | | |
| Atkins et al., 2016  Systematic review – narrative synthesis | Mixed – any interventions included. | Anti- TNFs (adalimumab, infliximab), ARA 290, neurostimulants (methylphenidate, armodafinil) | Adalimumab, infliximab, methylphenidate and armodafinil significantly reduced fatigue.  ARA290 (a novel molecule aimed at treating small-fibre neuropathy) showed no significant difference to control. | Anti-TNFs and neurostimulants may be effective treatments for reducing fatigue. |
| Sjogren’s Syndrome | | | | |
| Seneviratne et al., 2014  Conference abstract.  Systematic review – narrative synthesis. | Biologic or pharmacological interventions | Rituximab, epratuzumab, LEF, infliximab, zidovudine, dehyrdoepiandrosterone (DHEA), HCQ, anakinra, doxycycline | Rituximab, epratuzumab, LEF and zidovudine significantly reduced fatigue.  Infliximab effectiveness was mixed.  DHEA reduced fatigue but so did placebo.  HCQ, anakinra, doxycycline did not to reduce fatigue. | Low level of evidence for treating fatigue pharmacologically, but B cell directed therapies (e.g. rituximab) may be effective in treating fatigue. |
| Mixed - Neurological Disorders | | | | |
| Sheng et al., 2013  Systematic review – meta-analysis  Parkinson’s, Multiple Sclerosis, Traumatic Brain Injury, Post-polio syndrome (PPS) | Modafinil | Modafinil | Modafinil significantly reduced fatigue in TBI (SMD= -0.82, 95% CI -1.54, 0.11).  Modafinil has no significant effect on fatigue in Parkinson’s (SMD= -0.22, 95% CI -1.23, 0.79) or MS (SMD= -6.56, 95% CI -19.67, 6.55).  Modafinil has no significant effect on fatigue in PPS (narrative - 1 RCT). | Inconsistent results for modafinil across neurological conditions means there is not sufficient evidence to recommend it for fatigue treatment. |
| HIV | | | | |
| Jong et al. 2010  Systematic overview, including treatment of fatigue. | Mixed – any interventions included. | Fluoxetine, testosterone cypionate, DHEA, recombinant human growth hormone (rhGH), Psychostimulants (dextroamphetamine, methylphenidate hydrochloride, pemoline, modafinil). | Testosterone and psychostimulants significantly reduced fatigue.  Fluoxetine, DHEA and rhGH did not reduce fatigue. | Evidence for fatigue management using medication is not strong, but psychostimulants and testosterone may be effective. |
| Parkinson’s Disease | | | | |
| Bruno & Sethares, 2015  Integrative review including fatigue treatment.  (study overlap with Franssen et al. and Elbers et al.) | Mixed – any interventions included. | Methylphenidate, modafinil, rasagiline | Methylphenidate and rasagiline significantly reduced fatigue.  Modafinil not effective in reducing fatigue. | Methylphenidate and rasagiline may be effective for treating fatigue. |
| Elbers et al., 2015  Systematic review- narrative synthesis and meta-analysis | Mixed – any interventions included. | Levodopa-carbidopa, memantine, rasagiline, caffeine, methylphenidate, modafinil, doxepin | Insufficient evidence to determine whether doxepine reduces fatigue (one study, N=12, SMD = -1.50, 95% CI -2.84, -0.15).  Rasagiline reduced fatigue (one study, N = 1176, SMD = -0.27, 95% CI -0.39 to -0.16).  No significant effect for any of the other medications.    Methylphenidate reported not to be effective but same study as in Bruno review and paper reports significant improvement in fatigue.  **Subgroup analyses**  Trend: Modafinil more effective for people with underlying depression (SMD = -0.40, 95% CI -1.43, 0.62) than non-depressed (SMD = -0.08, 95% CI -0.73, 0.56). | Rasagiline may be effective for treating fatigue. |
| Franssen et al., 2014  Systematic review – narrative synthesis and meta-analysis.  (study overlap with Bruno & Sethares and Elbers et al.) | Mixed – any interventions included. | Amphetamines: Modafinil, methylphenidate.  Dopamines: pergolide mesilate, pramipexole, doxepin, rasagiline, levodopa, acute tryptophan depletion, memantine, caffeine. | Rasagiline (overlap with Bruno & Sethares). Doxepin (overlap with Elbers et al.). Methylphenidate and modafinil (overlap with Elbers et al. and Bruno & Sethares).  Pergolide mesilate and pramipexole shown to be significantly more effective than control/alternative.  Meta-analysis of amphetamines showed no significant effect on fatigue. | Reporting issues and study overlap with other Parkinson’s reviews means few novel conclusions can be drawn.  Pergolide mesilate and pramipexole may be effective in reducing fatigue. |
| Traumatic Brain Injury | | | | |
| Cantor et al., 2014  Systematic review – narrative synthesis | Mixed – any interventions included. | Modafinil, oral creatine, monoamergic stabiliser (-)-OSU6162, methylphenidate, donepezil, pramipexole, piracetam | Methylphenidate significantly reduced fatigue, but studies underpowered.  Modafinil and pramipexole did not reduce fatigue.  Evidence for piracetam, donepezil, creatine, monoamergic stabiliser is unclear/inconclusive. | Insufficient evidence to recommend any of these treatments for fatigue. |
| Mixed - Palliative care | | | | |
| Mücke et al., 2015  Systematic review – narrative synthesis (and meta-analysis).  MS, HIV/AIDS, post-polio, Parkinson's, End stage renal, ALS, multi, COPD  (study overlap with reviews of individual conditions)  (not including cancer related findings, n=18) | Pharmacological interventions | Amantadine, pemoline, methylphenidate, dexamphetamine, acetyl-L-carnitine (ALCAR), testosterone,  modafinil, fluoxetine, alfacalcidol, armodafinil, acetylsalicylic acid. | Amantadine, methylphenidate, carnitine, acetylsalicylic acid, dexamethasone,  alfacalcidol, armodafinil and modafinil reduced fatigue in a few studies, but weak and inconclusive evidence.  Heterogeneity in included samples made summarising findings difficult – also, overlap to other reviews focusing on a specific health condition. | Some positive results but the review found no evidence to support the use of a specific drug to treat fatigue in palliative care patients. |
| Systemic lupus erythematosus | | | | |
| Yuen & Cunningham, 2014  Systematic review – narrative synthesis | Mixed – any interventions included. | N-acetylcysteine (NAC), dehyrdoepiandrosterone (DHEA), belimumab | NAC and belimumab significantly reduced fatigue.  DHEA did not reduce fatigue. | NAC (2.4g/day may be an effective treatment of fatigue – minimal side effects. Belimumab may also be effective but side effects and costs need consideration. |
| Post-stroke | | | | |
| Wu et al., 2015  Systematic review – meta-analysis | Mixed – any interventions included. | Fluoxetine, duloxetine, enerion, (-)-OSU6162, citicoline, Chinese herbs combination, tirilazad mesylate. | Pharmacological interventions showed marginal significant effect for reducing fatigue (SMD = -1.23, 95% CI -2.40 to -0.06).  Anti-depressants (fluoxetine, duloxetine), and ((-)-OSU6162) (psychostimulant) did not reduce fatigue. | Insufficient evidence for the efficacy of any of these interventions for fatigue.  (Unclear reporting) |
| Chronic Fatigue Syndrome | | | | |
| Cleare et al., 2015  Systematic review – narrative synthesis | Mixed  Anti-depressants and corticosteroids  (and CBT and GET) | Fluoxetine,  Fludrocortisone, Hydrocortisone, Hydrocortisone + fludrocortisone | Fluoxetine, fludrocortisone and hydrocortisone did not significantly reduce fatigue. | No evidence for the effectiveness of these treatments for fatigue. |
| Peripheral neuropathy | | | | |
| White et al. 2014  Systematic review – narrative synthesis | Any interventions could be included but only pharmacological studies identified. | Amantadine  Ascorbic acid | Neither amantadine nor ascorbic acid significantly reduced fatigue. | No evidence to support the use of these medications for fatigue management. |
| Multiple Sclerosis | | | | |
| Asano et al. 2014  Systematic review – meta-analysis  (study overlap with other MS reviews) | Mixed – exercise, education and medication. | Amantadine, modafinil | Pharmacological interventions did not to reduce fatigue (SMD=0.07, 95%CI: −0.22, 0.37). Only one of seven studies reported significant intervention effect. | Weak and inconclusive evidence of pharmacological intervention for fatigue. |
| Branas et al., 2000  Scoping review… | Scoping review – any.  Systematic review – specific to amantadine and pemoline | Amantadine, pemoline, potassium channel  blockers (4-aminopyridine, 3,4,diaminopyridine) and antidepressants identified.  Amantadine and pemoline | Only amantadine and pemoline investigated further in systematic review as potentially effective.  Amantadine and pemoline did not to significantly reduce fatigue. | No consistent evidence for either drug to be recommended for fatigue. |
| Branas et al. 2000  …followed by systematic review – narrative synthesis  (study overlap with other MS reviews) |  |  |  |  |
| Brown et al. 2010  Systematic review - narrative synthesis | Pharmacological – specifically Modafinil | Modafinil | Inconsistent results  Modafinil reduced fatigue in four uncontrolled studies, but mixed findings in controlled studies – 1 of 2 reported fatigue reduction. | Modafinil may be a reasonable therapeutic option. |
| Khan et al. 2014  Systematic overview  (includes other reviews - overlap) | Mixed – any interventions included. | Amantadine, modafinil, pemoline | Cites Asano et al. (2014), Branas et al. (2000) and Peuckmann et al. (2010) (earlier version of Mücke et al. (2015)). | Insufficient evidence to support these pharmacological agents for MS-fatigue management. |
| Lee et al. 2008  Systematic review – narrative synthesis  (study overlap with other MS reviews) | Mixed – any interventions included. | Amantadine, pemoline, prokarin, modafinil, aspirin, pain medications.  (interferon-beta-1b – treatment related fatigue as a side effect of medication) | Prokarin and aspirin significantly reduced fatigue. Mixed findings for pemoline, amantadine and modafinil. | Prokarin and aspirin may be promising but evidence for any one drug (or drug interventions as a whole) as a treatment for fatigue, is limited. |
| Pucci et al. 2009  Systematic review – narrative synthesis  (study overlap with other MS reviews) | Pharmacological – specifically Amantadine | Amantadine | Small, inconsistent reduction in fatigue was reported. | No evidence supporting use of amantadine in fatigue management. |
| Tejani et al. 2012  Systematic review – narrative synthesis  (study overlap with other MS reviews) | Pharmacological – specifically Carnitine | Carnitine (compared to amantadine) | No significant difference in number of patients with improved fatigue between the two.  Absolute reduction in fatigue was significant in favour of carnitine. | Insufficient evidence to determine if carnitine has positive impact on fatigue (need an alternative control group). |
