# Supplementary material for: Fatigue interventions in long term, physical health conditions: A scoping review of systematic reviews
Source: PLoS One. 2018 Oct 12;13(10):e0203367. doi: 10.1371/journal.pone.0203367 (PMC6193578; doi:10.1371/journal.pone.0203367)
Supplement: S3 Table — (DOCX) [file pone.0203367.s005.docx]

**S3 Table. Reviews including exercise interventions for fatigue treatment.**

| **Reference** | **Focus of review** | **Intervention details** | **Summary of findings** | **Implications** |
| --- | --- | --- | --- | --- |
| Chronic Fatigue Syndrome | | | | |
| Cleare et al. (2015)  Systematic review – narrative synthesis. Clinical Evidence summary. | Mixed  GET (and CBT and anti-depressants and corticosteroids) | Graded Exercise Therapy (GET)  Aerobic (e.g. walking, cycling), one included progressive resistance.  15-40minutes, 1-5x week. | GET programmes reduce fatigue compared to control interventions. | GET may be an effective strategy for reducing fatigue. |
| Marques et al. (2015)  Systematic review – meta-analysis  (study overlap – majority of studies included in Cleare et al.) | Behavioural interventions with a graded physical activity component | GET, GET + drug placebo, pragmatic rehabilitation, CBT with graded exercise, Multi-convergent therapy and medication.  Variety of sessions: 8-14 total sessions, 12-24 weeks. | Interventions with a graded activity/exercise component had significant effect on fatigue post-treatment (Hedges g=0.61; 95% CI 0.44 to 0.78) and at follow-up (Hedges g=0.66; 95% CI 0.38 to 0.93).  **Subgroup** **analyses**  Interventions conducted in secondary–tertiary settings, interventions delivered by psychologists or psychotherapists, and interventions providing minimal contact (versus more intensive contact), reduced fatigue more.  Overall, interventions containing a psychological component and allowing flexibility in physical activity levels/goals reported to reduce fatigue more. | GET may be an effective strategy for reducing fatigue (as above).  To maximise improvement consideration should be given to: setting, facilitator, amount of contact, and making the intervention flexible with a psychological component. |
| Larun et al. (2016)  Systematic review – meta-analysis  (study overlap – all studies are included in Marques et al.) | Exercise therapy | Exercise therapy  Various: aerobic (walking, swimming, cycling, dancing at mixed levels of intensity). 12-26 weeks, 3-5 times per week, 5-15 minutes. Intensity ranged from low to high. | Exercise therapy significantly reduced fatigue (Standard mean difference (SMD)= -0.68; 95% CI -1.02, -0.35). Greater fatigue reduction compared to supportive listening and pacing.  No difference in fatigue reduction when exercise therapy compared to CBT.  **Subgroup analyses**  GET versus treatment as usual showed increased reduction in fatigue compared to anaerobic exercise versus relaxation.  No difference between GET and self-pacing.  No significant effect of diagnostic criteria or type of control group. | Exercise therapy may be an effective strategy for reducing fatigue. The exercise therapy seems to be more effective than pacing but similar in effect to CBT. |
| Castell et al. (2011)  Systematic review – meta-analysis  (study overlap – majority included in Cleare et al. or Marques et al.) | Mixed  CBT and GET | Graded Exercise Therapy  All individual in secondary care. | GET significantly reduced fatigue (hedges g= 0.41, CI = 0.06, 0.77) (similar to CBT).  GET appears to be more effective when started at low levels of intensity. | GET may be an effective treatment for fatigue, especially if started at low levels.  CBT may be more effective for treating patients with comorbid depression and anxiety. |
| Rheumatoid Arthritis | | | | |
| Cramp et al. (2013)  Systematic review – meta-analysis | Non-pharmacological interventions | Physical activity including pool based-therapy, yoga, dynamic strength training, aerobics, Tai Chi. | Physical activity significantly reduced fatigue  (SMD= -0.36, 95%CI -0.62 to -0.10). | Physical activity may be an effective intervention for fatigue. |
| Systemic Lupus Erythematosus | | | | |
| Del Pino-Sedeno et al. (2016)  Systematic review – narrative synthesis | Non-pharmacological interventions | Exercise  Aerobic in all, include strengthening in one. 8weeks – 8 months, typically 3x per week, 30-60 minutes. | Exercise significantly reduced fatigue in all studies (effect size (ES) ranged from 0.30 – 0.89). | Exercise may be beneficial for reducing fatigue. |
| Yuen & Cunningham (2014)  Systematic review – narrative synthesis  (study overlap with Del Pino-Sedeno et al.) | Mixed – any interventions included | Exercise  Primarily aerobic, 3x per week, 30-50 minutes, 8 to 12 weeks. | All but one study (6/7) reported that exercise significantly reduced fatigue. | Exercise may be beneficial for reducing fatigue. |
| Cleanthous et al.(2012)  Integrative review with specific section on interventions  (study overlap – all studies included in Del Pino-Sedeno et al. or Cleanthous et al.) | Non-pharmacological | Exercise  Graded exercise | Findings were inconsistent (3 of 5 studies showed a significant reduction in fatigue). | Exercise may be beneficial for reducing fatigue. |
| Parkinson’s Disease | | | | |
| Elbers et al. (2015)  Systematic review – meta-analysis | Mixed – any interventions included | Exercise  Two studies. Aerobic (treadmill) and aerobic/muscle strength/flexibility. 4-7 sessions per week. 12 weeks. | Exercise did not significantly reduce fatigue (SMD = -0.45, 95% CI -1.21 to 0.32). | No evidence that exercise is a beneficial intervention for fatigue. |
| Franssen et al. (2014)  Systematic review – narrative synthesis (and meta-analysis).  (study overlap) | Mixed – any interventions included | Behavioural  Exercise 1 study (overlap) | Exercise did not significantly reduce fatigue. | No evidence that exercise is a beneficial intervention for fatigue. |
| Bruno & Sethares (2015)  Integrative review with specific section on interventions to minimise fatigue.  (study overlap) | Mixed – any interventions included | Physical activity  1 intervention study (overlap)  1 cross-sectional study | Inconsistent findings  Intervention study – physical activity did not reduce fatigue. Cross-sectional study reports less fatigue in those who have higher levels of physical activity. | No evidence that exercise is a beneficial intervention for fatigue. |
| Traumatic Brain Injury | | | | |
| Cantor et al. (2014)  Systematic review – narrative synthesis | Mixed – any interventions included | Physical activity including aquatic exercise, tai chi, cardiorespiratory fitness, therapeutic recreational programme.  5 days to 12 weeks. 2-3x week. | Physical activity-orientated interventions have little effect on fatigue. | No evidence that exercise is a beneficial intervention for fatigue. |
| Post-stroke | | | | |
| Wu et al. (2015)  Systematic review – narrative synthesis (1 meta-analysis for pharma v. non-pharma). | Mixed – any interventions included | Physical training  Cognitive behavioural therapy + graded activity training. Aerobic (cycling, walking).  1-5x week, 6weeks to 6months. | CBT + graded activity reduced fatigue more than CBT alone, but not significantly so. | Insufficient evidence to judge whether physical activity with psychological interventions may improve fatigue. |
| End Stage Kidney Disease | | | | |
| Astroth et al. (2016)  Systematic review – narrative synthesis | Non-pharmacological interventions | Exercise  Majority aerobic (walking, cycling). Also yoga, physical therapy (stretching, strengthening).  Varying durations and frequencies. | Inconsistent findings.  Majority of exercise studies reporting non-significant reduction in fatigue.  Those which reported significant effects of exercise included cycling and yoga. | Limited evidence to suggest exercise is a beneficial intervention for fatigue. |
| Coronary Heart Disease | | | | |
| Puetz et al. (2006)  Systematic review – meta-analysis | Cardiac rehabilitation exercise programme | Cardiac rehabilitation exercise programmes or chronic exercise interventions (4+weeks). Multifactor or just exercise. | Cardiac rehabilitation exercise programs significantly reduced fatigue (d=0.59; 95% CI 0.47–0.71); clinically meaningful.  **Subgroup analyses**:  Non-controlled studies reported significantly larger reduction in fatigue (d=0.58) than controlled studies (d=0.32). Multifactor interventions reported larger effect (d=0.52) than exercise alone (d=0.34) but non-significantly so. | Cardiac rehabilitation/exercise programmes may be effective at reducing fatigue. |
| Inflammatory Bowel Disease | | | | |
| Artom et al. (2016)  Integrative review with specific section on managing fatigue. | Mixed – any interventions included. | One study on exercise advice (15 minute consultation) vs. omega-3 fish oil and dietary consultation and placebo. | Exercise advice significantly reduced fatigue impact. | Advising patients to engage in exercise may be an effective way to reduce the impact of fatigue. |
| Sarcoidosis | | | | |
| Atkins & Wilson (2016)  Systematic review – narrative synthesis | Mixed – any interventions included. | Physical training programmes including muscle resistance (progressive) and endurance training.  Typically 12 weeks. | Reduced fatigue reported in the included studies. | Physical training may be beneficial for fatigue. |
| Fibromyalgia | | | | |
| Russell et al. (2014)  Abstract  Systematic review – narrative synthesis | Exercise | Exercise interventions (details not given). | Exercise interventions that reduced fatigue were of moderate intensity (e.g. pool, yoga, walking), delivered more than once a week for 60minutes, for a mean duration of 10 weeks. | Exercise in certain forms seems to be beneficial in improving fatigue. |
| Mixed health populations | | | | |
| Smith & Hale (2007)  Overview article – narrative synthesis.  MS, Parkinson’s, HIV/AIDS, (cancer)  (study overlap) | Non-pharmacological | Exercise  Aerobic, resistance, exercise + other treatment combinations.  Alternative medicine  Tai Chi, Yoga  (few studies included). | MS – aerobic exercise did not significantly reduce fatigue (overlap).  HIV – one study found reduced fatigue after aerobic training and resistance training, but did not define measurement or use statistical significance so not formally included in review.  Effects of aerobic and resistance exercise in people with Parkinson’s and HIV/AIDS - unexplored.  (Increasing evidence that both aerobic and resistance exercise may reduce cancer fatigue during and following treatment.)  Tai Chi and Yoga potentially promising but inconsistent and incomplete results mean drawing conclusions is difficult. | Few conclusions can be drawn (study overlap). |
| Neill et al. (2006)  Systematic review – narrative synthesis  Mixed – MS, RA, SLE  (study overlap) | Non-pharmacological interventions | Exercise  Aerobic (walking, jogging, swimming), average 12 weeks, mostly 3x per week, 30-60minutes. | Inconsistent findings – conclusion reported that low impact aerobic exercise may be effective for some people. | Exercise may be effective in reducing fatigue in some people. |
| Multiple Sclerosis | | | | |
| Heine et al. (2015)  Systematic review – meta-analysis and narrative synthesis of those not included. | Exercise therapy | Exercise interventions  Categorised as endurance training (1-5x week, 3 weeks to 6 months, cycling, treadmill walking), muscle power training (2-5x week, 8 to 12 weeks), task-orientated training, mixed, other (e.g. yoga). | Exercise significantly reduced fatigue compared to ‘non-exercise’ control (SMD= -0.53, 95%  CI -0.73 to -0.33) and any control condition  (SMD= -0.35; 95% CI -0.57,-0.13).  **Subgroup analyses**  Significantly larger effect when comparing exercise to non-exercise controls (SMD= -0.58) than other exercise controls (SMD= 0.28).  Endurance (SMD= -0.43; 95% CI -0.69, 0.17), mixed training (SMD= -0.73; 95% CI -1.23, 0.23) and ‘other’ (SMD= -0.64; 95% CI -1.00, -0.29) significantly reduced fatigue.  Muscle power training and task orientated training did not significantly reduce fatigue.  No significant difference in fatigue reduction depending on fatigue measure (MFIS, FSS, Other).  No intervention for risk of relapse. | Exercise may be an effective treatment of fatigue. Endurance, mixed or other training may be more beneficial than muscle power or task-orientated training. |
| Andreasen et al. (2011)  Systematic review – narrative synthesis  (study overlap) | Exercise therapy | Endurance training, resistance training, combined endurance and resistance, other training (aquatic exercise, walking, bicycle, upper body ergometer) | Inconsistent findings.  **Subgroup discussion**  Majority of studies with fatigued patients report significantly reduced fatigue, whereas studies with non-fatigued populations do not.  Few studies compare exercise modalities but of those that did, neither found differences between the interventions.  Conclusions not drawn about duration, frequency and intensity of exercise, or fatigue scale used.  Broached idea of socialisation – social interaction may also provide a benefit. | Exercise may be a promising technique for fatigue reduction, specifically for those patients who are actually fatigued. |
| Asano & Finlayson (2014)  Systematic review – meta-analysis  (study overlap – all elsewhere) | Exercise, Education, Medication | Exercise  Including aerobic, aquatic, and inspiratory muscle exercise; vestibular rehabilitation program; progressive resistance  training; climbing; and yoga | Exercise significantly reduced fatigue (Pooled ES = 0.57; 95% CI 0.10, 0.64) (larger than for amantadine and modafinil), (range -0.24 to 2.05).  Three of ten studies reported a significant intervention effect, after taking 95% CI into consideration. | Exercise may be effective in reducing fatigue in some patients (e.g. younger adults, non-progressive MS and those with mild to moderate disability). |
| Asano et al. (2015)  Scoping review  (study overlap – majority elsewhere) | Rehabilitation interventions | Exercise  Including progressive resistance training, resistance training, yoga or climbing, vestibular rehabilitation, home based elf-managed graded exercise, aquatic, maximal effort, elliptical, individualised program, inspiratory muscle timing, single session | Seventeen studies identified. Progressive resistance training most common (n=4).  **Subgroup discussion**  ES for the participants with fatigue was positive and significant, whereas ES for the participants without fatigue was negative and non-significant.  Common features of interventions that significantly reduced fatigue: minimum of six weeks, at least 2x per week for at least 45mins, trained interventionist. | Not sufficient evidence to make firm recommendations.  Authors suggested exercise interventions that progress over time, incorporate education and involve trained facilitators seem optimal. |
| Pilutti et al. (2013)  Systematic review – meta-analysis  (study overlap – all under Heine et al., 2015) | Exercise training | Various exercise interventions | Exercise training significantly reduced fatigue  (hedges g= 0.45, 95% CI 0.22, 0.68). | Exercise may be effective in reducing fatigue. |
| Latimer-Cheung et al. (2013)  Systematic review – narrative synthesis  (study overlap) | Exercise training | Protocols included aerobic exercise (arm/leg cycling), resistance training (weight machines, free weights, resistance bands), combined aerobic and resistance training, and a variety of other forms of physical activity (sport, yoga, body weights, supported treadmill training, aquatic exercise, FES cycling, Pilates). | Inconsistent evidence.  Effect of aerobic exercise mixed, majority reported no significant reduction in fatigue (9/13).  Resistive training significantly reduced fatigue.  Combined training significantly reduced fatigue.  Other exercises: 10/11 studies reported a significant reduction on at least one measure of fatigue. | Interventions including a resistance-training component may be most effective for reducing fatigue. |
| Khan et al. (2014)  Systematic overview  (study overlap) | Fatigue management | Physical modalities  (including Asano & Finlayson, 2014; Latimer-Cheung et al., 2013; Andreasen et al., 2012; Neill et al., 2006; individual exercise studies – aquatic therapy and tai chi - included elsewhere). | Inconsistent evidence.  Data for optimal type and intensity of exercise is insufficient.  Some types of exercise which include endurance and a resistance component may have potential benefits. | As above. |
| Godhrawala et al. (2010)  Abstract – narrative synthesis  (potential study overlap) | Resistance training | Resistance training alone (n=2), resistance + exercise therapy (n=6) | Inconsistent reporting of findings.  Resistance training alone did not significantly reduce fatigue. | Resistance training may be effective in reducing fatigue, if combined with another element of exercise therapy. |
| Branas et al. (2000)  Scoping review  (study overlap) | Mixed – any interventions included. | Aerobic exercise | One randomised controlled trial and one case series identified. | Limited research identified. |
