# Supplementary material for: Fatigue interventions in long term, physical health conditions: A scoping review of systematic reviews
Source: PLoS One. 2018 Oct 12;13(10):e0203367. doi: 10.1371/journal.pone.0203367 (PMC6193578; doi:10.1371/journal.pone.0203367)
Supplement: S4 Table — (DOCX) [file pone.0203367.s006.docx]

**S4 Table. Reviews including psychological/behavioural interventions for fatigue treatment.**

| **Reference** | **Focus of review** | **Intervention details** | **Summary of findings** | **Implications** |
| --- | --- | --- | --- | --- |
| Chronic Fatigue Syndrome | | | | |
| Cleare et al. (2015)  Systematic review – narrative synthesis. Clinical Evidence summary. | Mixed  Cognitive Behavioural Therapy (CBT)  (and GET and anti-depressants and corticosteroids) | CBT | CBT programmes reduce fatigue compared with control interventions. | CBT may be effective for reducing fatigue. |
| Castell et al. (2011)  Systematic review – meta-analysis  (study overlap – majority elsewhere) | Mixed  CBT and GET | CBT  Varied delivery: face-to-face, telephone. 6-16 hours. Group and individual settings. | CBT significantly reduced fatigue (Hedges g = 0.35; 95% CI 0.24, 0.46) (similar in effect to GET).  **Subgroup analyses**  Higher number of treatment hours significantly predicted greater fatigue reduction.  Diagnostic criteria, control group, treatment format, setting, study quality, pre-treatment illness duration and duration of intervention did not affect fatigue. | CBT may be effective in reducing fatigue.  To maximise improvement consider: treatment hours. |
| Malouff et al. (2008)  Systematic review – meta-analysis  (study overlap – all elsewhere) | CBT | CBT  Varied widely in intensity and specific therapeutic methods. | CBT significantly reduced fatigue (d=0.48, 95% CI 0.27, 0.69)  **Subgroup analyses**  Trend of increased fatigue reduction for physical fatigue compared to mental fatigue (d=0.81, d=0.20) and higher number of hours (d=0.47).  No significant effect of treatment type, format, control group, participant sample, diagnostic criteria, number of sessions, months of follow-up and study quality.  No significant difference between objective and subjective fatigue. | CBT may be effective in reducing fatigue.  To maximise improvement consider: hours of treatment, type of fatigue targeted. |
| Price et al. (2010)  Systematic review – meta-analysis  (study overlap) | CBT | CBT  (alone and combined with other interventions – biofeedback, relaxation, aerobic exercise).  Various: individual/groups, practitioner experience, frequent, duration.  Compared to usual care, other psychological therapies (relaxation, counselling, guided support, education + support) and exercise. | Compared to usual care, CBT significantly reduced fatigue after treatment (standard mean difference (SMD)= -0.39; 95%CI -0.60, -0.19) and at follow up (SMD - 0.47; 95%CI -0.69,-0.25).  **Subgroup analyses** (significance not stated)  Larger reduction in fatigue for individual care, comparison to treatment as usual (compared to waiting list) and CBT incorporating increased activity.  No difference between <8 weeks and >8 weeks duration.  Compared to other psychological therapies, CBT significant reduced fatigue after treatment  (SMD= -0.43, 95%CI -0.65, -0.20) and at follow up (SMD= -0.47, 95% CI -0.83, -.011).  CBT combined with other interventions significantly reduced fatigue compared to usual care.  No significant difference in fatigue reduction when CBT compared to exercise. | CBT may be effective in reducing fatigue.  To maximise improvement consider: delivery format (individual), and incorporating increased activity. |
| Systemic Lupus Erythematosus | | | | |
| Del Pino-Sedeno et al. (2016)  Systematic review – narrative synthesis | Non-pharmacological interventions | Behavioural and psychological approaches  CBT, psychoeducation, relaxation, self-management and counselling.  Number of session 1-36, typically 30-45mins (but also 2hrs), phone/home, various facilitators. | All studies reported significant fatigue reduction on at least one measure (effect sizes (ES) ranged from 0.24 – 0.84). | These behavioural and psychological approaches may be beneficial for reducing fatigue. |
| Yuen & Cunningham (2014)  Systematic review – narrative synthesis  (study overlap) | Mixed – any interventions included | Psychosocial interventions  CBT, psychoeducation, counselling, psychotherapy and biofeedback | Inconsistent evidence: Only 1 of 5 randomised controlled trials (RCTs) demonstrated fatigue reduction, 2 non-RCTs demonstrated reduced fatigue. | Evidence to support psychosocial interventions in alleviating fatigue in patients with SLE is very weak. |
| Cleanthous et al.(2012)  Integrative review with specific section on interventions  (study overlap – all but 1 included elsewhere) | Non-pharmacological | Psychological and self-management.  Self-management and counselling interventions (overlap), stress management + biofeedback (overlap), expressive writing. | Telephone counselling/education interventions, stress management programmes and expressive writing significantly reduced fatigue scores. | Findings suggest these interventions may be effective in reducing fatigue, but some inconsistent results (as above).  Expressive writing may be an effective technique for reducing fatigue. |
| Parkinson’s Disease | | | | |
| Franssen et al. (2014)  Systematic review – narrative synthesis | Mixed – any interventions included | Behavioural  Behavioural programme, CBT (2 studies) | Neither study reported significant reduction in fatigue. | No evidence that CBT/behavioural programmes are effective. |
| Traumatic Brain Injury | | | | |
| Cantor et al. (2014)  Systematic review – narrative synthesis | Mixed – any interventions included | Cognitive behavioural interventions  CBT, CBT for anxiety, CBTi, CBT + psychoeducation. | Inconsistent findings. | Little evidence that cognitive behavioural therapy is an effective treatment for fatigue. |
| Post-stroke | | | | |
| Wu et al. (2015)  Systematic review – narrative synthesis (one meta-analysis pharma v. non-pharma). | Mixed – any interventions included | Psychological interventions  Chronic disease self-management, group psychoeducation, mindfulness based stress reduction, cognitive behavioural therapy. | Unclear reporting but conclusion states that studies did not demonstrate effectiveness due to limitations in study design. | No evidence that CBT/behavioural programmes are effective. |
| End Stage Kidney Disease | | | | |
| Picariello et al. (2017)  Systematic review – meta-analysis | Social-psychological interventions. | CBT based (targeted sleep, fluid adherence, physical functioning), shared care and practical nurse support, community support, nutrition self-management, pharmaceutical care programme, educational + behavioural programme.  2-96 sessions, mostly 30-60minutes, mostly face-to-face, individual or group. | Social-psychological interventions significantly improved fatigue (SMD = 0.37; 95% CI 0.15, 0.59), comparable at short and longer term follow up. Individually, a third of the studies found significant improvement in fatigue.  **Sub-group analyses**  Stress management/relaxation interventions and those with less experienced facilitators were more effective at reducing fatigue.  No significant differences depending on CBT content, sample or comparison group. But fatigue reduction became non- significant for samples deemed non-fatigued at baseline and when comparing to active control group. | Overall, as an intervention category, social-psychological interventions seem to be effective in reducing fatigue but differences on an individual level require further exploration. |
| HIV | | | | |
| Jong et al. (2010)  Integrative review with a specific section on the treatment of fatigue. | Mixed – any interventions included | Psychological interventions  Cognitive-behavioural stress management with psychoeducation, and relaxation intervention. | Relaxation intervention significantly reduced fatigue (no significant difference to psychotherapy and non-psychiatric treatment).  Cognitive behavioural intervention did not significantly reduce fatigue. | Relaxation may be an effective fatigue reduction technique. |
| Rheumatoid Arthritis | | | | |
| Cramp et al. (2013)  Systematic review – meta-analysis | Non-pharmacological interventions | Psychosocial interventions – expressive writing, CBT, mindfulness, lifestyle management, energy conservation, self-management and group education. | Psychosocial interventions (as one group) significantly reduced fatigue (SMD= -0.24, 95% CI -0.40 to -0.07). | Psychosocial interventions as a whole may be effective in reducing fatigue, but components of effective treatment need to be clarified. |
| Multiple Sclerosis | | | | |
| Asano et al. (2015)  Scoping review  (study overlap) | Rehabilitation | Behaviour change interventions  Including energy conservation, CBT or group psychotherapy, multidisciplinary rehabilitation, mindfulness, fatigue management programme, education and counselling, memory training, community rehab, (cooling) | Twenty one studies identified. Energy conservation most common (n=8), followed by CBT (n=5). ES ranged from 1.25 – 2.99.  Nine studies reported significant reduction in fatigue. | Behaviour change interventions offer more flexibility than exercise interventions as participants are able to review their issues and develop skills to adjust routine, activities and environment at their own pace. |
| Branas et al. (2000)  Scoping review  (study overlap) | Mixed – any interventions included. | Behavioural advice.  Extended multidisciplinary outpatient rehabilitation | Evidence: established clinical practice.  One non-randomised controlled trial – seemed to show benefit on fatigue domain. | Very little research evidence identified. |
| Blikman et al. (2013)  Systematic review – narrative synthesis and meta-analysis  (study overlap – all identified in Asano et al. 2015 scoping review) | Energy conservation | Energy conservation management (ECM) programmes | ECM programmes significantly reduced fatigue compared to support group (mean difference (MD)= 23.80; 95% CI -45.65, -1.95) and waiting list controls (Cognitive scale: MD= 2.91; 95% CI -4.32 to -1.50. Physical scale (MD=2.99; 95% CI -4.47 to -1.52. Psychosocial scale (MD=6.05; 95% CI -8.72 to -3.37) (but limited evidence due to low number of studies, n=2 in each).  ECM-related fatigue treatments did not significantly reduce fatigue. | ECM based on Packer’s programme specification may be effective for reducing fatigue. |
| Khan et al. (2014)  Systematic overview  (study overlap) | Mixed – any interventions included | Behavioural and educational interventions (cite Asano & Finlayson, and Neill).  Fatigue management programmes, energy conservation (cite Blikman), mindfulness (cites another non-fatigue focus systematic review), cognitive and psychological interventions (overlap) | Majority of overlapping studies.  Fatigue management programme significantly reduced fatigue severity at 1, 4 and 12 months, and mindfulness reduced fatigue, maintained at 6 months. | Fatigue management programmes and mindfulness may be effective fatigue management strategies. |
| Lee et al. (2008)  Systematic review – narrative synthesis  (study overlap) | Mixed – any intervention included | Energy conservation | Energy conservation course significantly reduced fatigue. | See above. |
| Kohli et al. (2012)  Poster. Systematic review – meta-analysis  (study overlap) | Cognitive behavioural therapy | CBT | CBT significantly reduced fatigue compared to no therapy (weighted mean difference (WMD)= -7.04), relaxation therapy (WMD= -4.29 and follow up WMD= -2.74), and supportive expressive group therapy (WMD= -12.2). | CBT may be effective for reducing fatigue, possibly more so than other approaches. |
| Van den Akker et al. (2016)  Systematic review – meta-analysis | Cognitive behavioural therapy | CBT  Five solely CBT, 1 CBT components.  All solely CBT addressed activity scheduling, dealing with stressors, unhelpful thoughts, apply skills, relapse plan. Most also looked at understanding, sleeping, emotions, symptom focussing.  Heterogeneity: delivery, intensity, therapists, control conditions. | CBT significantly reduced fatigue after treatment (SMD=-0.47; 95% CI -0.88 to -0.06) and at follow up (SMD = -.30; 95%CI -0.51, -0.08).  Inconsistencies for follow up - one study reported a reduction in treatment effect, one remained and one got stronger. | CBT may be effective for reducing fatigue in the short and long term (with the effect reducing over time). |
| Wendenbourg et al. (2017)  Systematic review – meta-analysis  (study overlap) | Patient education | CBT-based, energy conservation, multi-disciplinary self-management, mindfulness.  Group and individual programs, personal/online/telephone, 4-16 week duration. | Educational interventions significantly reduced fatigue severity (SMD= -0.43; 95% CI -0.74 to -0.11) and fatigue impact (SMD= -0.48; 95%CI -0.82, -0.15).  **Subgroup** **analyses**  CBT-based interventions (SMD= -0.60; 95% CI -1.08, -0.11) reduced fatigue severity more than non-CBT interventions (SMD= -0.20; 95% CI -0.60, 0.19).  Individual approaches (SMD= -0.80; 95% CI  -1.13, -0.47) reduced fatigue severity more than group approaches (SMD= -0.17; 95% CI -0.39, 0.05). | Educational interventions may be effective for reducing fatigue severity.  To optimise intervention consider: incorporating CBT elements, individual approaches. |
| Inflammatory Bowel Disease | | | | |
| Artom et al. (2016)  Integrative review with specific section on managing fatigue. | Mixed – any interventions included. | Psychosocial interventions  Problem solving therapy, solution focused therapy, stress management (professional led and self-led) | Solution focussed therapy significantly reduced fatigue at 3 months, but not at 9 months.  Small, non-significant reduction in fatigue for stress management. | Solution focussed therapy may be a promising intervention for fatigue. |
| Mixed health conditions | | | | |
| Neill et al. (2006)  Systematic review – narrative synthesis  Mixed – MS, RA, SLE  (study overlap) | Non-pharmacological interventions | Behavioural strategies  Educational interventions (group self-management, energy conservation, stress management, active living, self-care), rehabilitation, counselling/CBT | Behavioural interventions appeared effective in reducing fatigue but inconsistent findings. | Inconsistent results mean drawing conclusions difficult. |
| Smith & Hale (2007)  Overview article – narrative synthesis  MS, Parkinson’s, HIV/AIDS, (cancer)  (study overlap) | Mixed – any interventions included | Energy conservation  (MS and cancer studies). | Significant reduction in fatigue for intervention group. | Findings suggest energy management programmes may be effective in reducing fatigue |
| Ulrichsen et al. (2016)  Systematic review – meta-analysis  Post-stroke, traumatic brain injury and MS | Mindfulness | Mindfulness  (including body scanning, yoga, weekly classes, homework, classes).  All 8 weeks. | Mindfulness significantly reduced fatigue  (ES= -0.37; 95% CI -0.58, -0.17).  **Subgroup discussion**  Strongest effects seen in the studies which used fatigue cut-offs for recruitment and used mental fatigue outcome. | Mindfulness interventions may be effective in reducing fatigue, possibly more so for severely fatigued individuals/those reporting mental fatigue. |
| Menzies & Jallo (2011)  Systematic review – narrative synthesis  Asthma, bronchitis/emphysema, cancer, congestive heart failure, MS, HIV | Guided Imagery | Guided Imagery  Study length ranged from 1 session - 6 weeks. Duration ranged from 10 - 30. Frequency of use ranged from 1 to 5-7 times. Variable protocols and details of images. | Inconsistent findings.  Significant reduction in fatigue for studies with: greatest total duration of exposure, targeted imagery, and >30 participants. | Guided imagery may be an effective strategy, if it is tailored to the focus of the fatigue intervention. |
