# Supplementary material for: Fatigue interventions in long term, physical health conditions: A scoping review of systematic reviews
Source: PLoS One. 2018 Oct 12;13(10):e0203367. doi: 10.1371/journal.pone.0203367 (PMC6193578; doi:10.1371/journal.pone.0203367)
Supplement: S5 Table — (DOCX) [file pone.0203367.s007.docx]

**S5 Table. Reviews including complementary medicine non-pharmacological interventions for fatigue treatment.**

| **Reference** | **Focus of review** | **Intervention details** | **Summary of findings** | **Implications** |
| --- | --- | --- | --- | --- |
| Rheumatoid Arthritis | | | | |
| Cramp et al. (2013)  Systematic review – narrative synthesis | Non-pharmacological interventions | Other, non-pharmacological interventions.  Herbal medicine (andrographis paniculata), Mediterranean diet, omega-3 fatty acid supplementation, reflexology, provision of health tracker information. | Mediterranean diet significantly reduced fatigue compared to Western diet (no change)  Omega-3 significantly reduced fatigue (but control group also improved).  Reflexology – reduction in fatigue reported but no inferential statistics.  No significant fatigue reduction for health tracker information and herbal medicine. | Mediterranean diet may be effective in improving fatigue. |
| Systemic lupus erythematosus | | | | |
| Yuen & Cunningham (2014)  Systematic review – narrative synthesis | Mixed – any interventions included | Diet manipulation (low glycaemic index diet and low calorie diet), vitamin D supplementation, UV phototherapy, acupuncture. | Phototherapy significantly reduced fatigue.  Both diets and acupuncture/minimal needling significantly reduced fatigue, but did not reach minimal clinically important difference.  Inconsistent findings for vitamin D supplementation. | Phototherapy may be effective for reducing fatigue.  Diet and acupuncture may also have an effect, but clinical relevance was questionable. |
| Del Pino-Sedeno et al. (2016)  Systematic review – narrative synthesis  (study overlap) | Non-pharmacological interventions | Acupuncture (and minimal needling)  Diets – low glycaemic index diet, calorie-restricted diet.  Phototherapy (UV radiation) | Significant reduction in fatigue reported for all; acupuncture and minimal needling, both diets and phototherapy (different trial to other review). | Acupuncture, minimal needling, diet manipulation and phototherapy may be effective in reducing fatigue. |
| Inflammatory Bowel Disease | | | | |
| Artom et al. (2016)  Integrative review with specific section on managing fatigue. | Mixed – any interventions included. | Omega-3 fish oil v. exercise advice v. dietary consultation v. placebo | Omega-3 fish oil significant worsened fatigue scores. | Omega-3 fish oil seems to be detrimental in reducing fatigue. |
| Mixed health conditions | | | | |
| Neill et al. (2006)  Systematic review – narrative synthesis  MS, RA, SLE  (study overlap) | Non-pharmacological interventions | Nutritional supplements  Acetyl-carnitine (overlap with Tejani – under pharmacological)  Fish oil (overlap with Cramp)  Physiological approaches: pulsed electromagnetic field (EMF), body cooling (overlap with Lee and Khan). | Acetyl-L-carnitine reduced MS fatigue and was significantly more effective than amantadine.  Fish oil increased time to fatigue onset in RA.  EMF wearable devices significantly reduced fatigue, but mattresses were not (MS).  Cooling significantly reduced fatigue (MS). | These supplements may be effective in reducing fatigue.  Electromagnetic field devices and cooling may be effective strategies for reducing fatigue. |
| Multiple Sclerosis | | | | |
| Lee et al. (2008)  Systematic review – narrative synthesis | Mixed – any interventions included | Cooling (1hr per day, 1 month), pulsed electromagnetic therapy (overlap) (portable, daily exposure, 4 weeks). | Significantly reduced fatigue after cooling and pulsed electromagnetic therapy. | Cooling and pulsed electromagnetic therapy may be effective in reducing fatigue. |
| Khan et al. (2014)  Systematic overview | Mixed – any interventions included | Cooling and pulsed electro-magnetic devices. | Significantly reduced fatigue after cooling and pulsed electromagnetic therapy. | Cooling and pulsed electro-magnetic devices may be effective in reducing fatigue. |
| Branas et al. (2000)  Scoping review  (study overlap) | Mixed – any interventions included. | Cooling systems.  Alternative medications (bee venom, cannabis/cannibinoids, acupuncture/acupressure, yoga)  Pulsing magnetic field | Anecdotal evidence  Anecdotal evidence  1 randomised controlled trial | Limited research identified. |
| Traumatic Brain Injury | | | | |
| Cantor et al. (2014)  Systematic review – narrative synthesis | Mixed – any interventions included | Other interventions  Electroencephalographic biofeedback, cranial electrotherapy stimulation, bright blue light treatment. | Significant reduction in fatigue after electroencephalographic biofeedback (general and mental fatigue), cranial electrotherapy stimulation and blue light treatment. | Electroencephalographic biofeedback, cranial electrotherapy stimulation and bright blue light treatment may be promising for reducing fatigue. |
| Chronic Fatigue Syndrome | | | | |
| Adams et al. (2010) | Traditional Chinese medicinal herbs | No studies met inclusion criteria | n/a | none |
| Wang et al. (2014)  Systematic review – meta-analysis | Traditional Chinese medicine | Chinese herbal medicine, acupuncture, electronacupuncture, moxibustion, acupoint application, Qigong | Chinese herbal medicine significantly reduced fatigue according to meta-analysis (mean difference (MD)= -5.93; 95%CI -7.51, -4.34) and individual studies.  Acupuncture significantly reduced total fatigue  (MD= -2.61, 95% CI -3.56, -1.65).  Individual studies reported that electroacupuncture, moxibustion, acupoint application and Qigong significantly reduced fatigue. | Traditional Chinese medicine may be effective in alleviating fatigue symptoms. |
| Mixed health conditions | | | | |
| Smith & Hale (2007)  Overview article – narrative synthesis  MS, Parkinson’s, HIV/AIDS, (cancer) | Mixed – any interventions included | Complementary and alternative medicine  Acupuncture, Tai Chi, Yoga | Preliminary findings provide some positive indications. | Inconsistent and incomplete results mean drawing conclusions is difficult |
| Post-stroke | | | | |
| Wu et al. (2015)  Systematic review – narrative synthesis (plus one meta-analysis for pharma v. non-pharma). | Mixed – any interventions included | Traditional Chinese medicine: herbs and electroacupuncture + cupping  CPAP | Unclear reporting – conclusions state Chinese medicines are promising.  Continuous positive airway pressure not effective for reducing fatigue. | Traditional Chinese medicine techniques may be effective for reducing fatigue. |
| End Stage Kidney Disease | | | | |
| Astroth et al. (2016)  Systematic review – narrative synthesis | Non-pharmacological interventions | Eastern medicine (acupressure, acupuncture, far infra-red ray acupoint, transcutaneous electric acupoint stimulation)  Occupational therapy | All ‘Eastern medicine’ techniques shown to significantly reduce fatigue.  Occupational therapy did not. | The Eastern medicine techniques described may be effective methods for reducing fatigue. |
| Parkinson’s Disease | | | | |
| Bruno & Sethares (2015)  Integrative review with specific section on fatigue interventions | Mixed – any interventions included | Alternative interventions  Japanese massage (one off, 30 minutes) | Fatigue scores decreased after the massage. | Japanese massage may alleviate fatigue in the short term. |
