# Supplementary material for: Fatigue interventions in long term, physical health conditions: A scoping review of systematic reviews
Source: PLoS One. 2018 Oct 12;13(10):e0203367. doi: 10.1371/journal.pone.0203367 (PMC6193578; doi:10.1371/journal.pone.0203367)
Supplement: S6 Table — (DOCX) [file pone.0203367.s008.docx]

**S6 Table. Excluded reviews.**

| **Authors and year** | **Condition and Intervention** | **Reason for Exclusion** |
| --- | --- | --- |
| Anonymous (2007) | Chronic fatigue syndrome (CFS)  Any | Primary outcome of review not specifically fatigue; all outcomes considered. |
| Almeida et al. (2010) | Rheumatoid arthritis (RA)  Non-biologic pharmacological interventions | Protocol |
| Balliesio et al. (2014)  Poster | Insomnia  CBTi | Insomnia a set of symptoms, not a chronic physical health condition. |
| Balsamo et al. (2014) | RA  Exercise | Focus of review was fatigue assessment rather than management. |
| Boehm et al. (2012) | Mixed  Yoga | Mixed population - predominantly cancer and also including healthy participants. |
| Campbell et al. (2009)  Conference abstract | RA  Biological vs DMARDS (pharmacological) | Emailed author for full text/further information to make decision for inclusion – primary outcome of review unclear, but no response. |
| Caceres et al. (2008) | Multiple sclerosis (MS)  Non-pharmacological | Protocol |
| Chambers et al. (2006) | CFS  Any | Primary outcome of review not specifically fatigue; all outcomes considered. |
| Chester et al. (2006) | Chronic rhinosinusitis  Endoscopic sinus surgery | Surgical intervention. |
| Cho et al. (2005) | CFS  Placebo response | Primary outcome of review not specifically fatigue; fatigue included as possible outcome but not necessary. |
| Collatz et al. (2016) | CFS  Drug therapies | Primary outcome of review not specifically fatigue; outcomes not specified. |
| Czuber-Dochan et al. (2013) | Inflammatory bowel disease (IBD)  Any | Artom et al. (2016) updates search and findings. |
| Edmonds et al. (2013) | CFS  Exercise | Larun et al. (2016) updates this paper. |
| Farrell et al. (2015) | IBD  Any | Protocol |
| Griffith et al. (2008) | CFS  Any | Not systematic review process – review article. |
| Heesen (2016) | MS  Any | Full text couldn’t be found (author, library) |
| Hicks et al. (2008) | Traumatic brain injury (TBI)  Any | Protocol |
| Ibitoye et al. (2016) | Spinal cord injury  Functional electrical stimulation | Treatment associated fatigue management. |
| Immink et al. (2014) | Neurological disorders  Self-regulation and mindfulness | Not systematic review process – overview article. Search and inclusion criteria not systematic. |
| Ishaque et al. (2012) | Mixed  Rhodiola rosea (herbal medicine) | All but one population are healthy participants. |
| Johansen et al. (2012) | End stage kidney disease (ESKD)  Erythropoiesis-stimulating agents | Managing fatigue is not the focus of the review, merely documenting its improvement as a side effect of anaemia treatment. |
| Kim et al. (2013) | CFS  Complementary and Alternative Medicine | Primary outcome of review not specifically fatigue; outcomes not clearly stated. |
| Knight et al. (2013) | CFS  Any | Primary outcome of review not specifically fatigue; any clinical outcomes. |
| Lam et al. (2007) | Depression  Modafinil | Depression – not physical health condition. |
| Lee et al. (2011) | Mixed  Foot reflexology | Population includes healthy participants.  ‘Patients’ not described. |
| Luctkaar-Flude et al. (2015) | Mixed  Neurofeedback | Population includes healthy participants. |
| McGeough et al. (2009) | Post-stroke  Any | Wu et al. (2015) updates this paper. |
| Mulrow et al. (2001) | CFS  Any | Primary outcome of review not specifically fatigue; outcomes not specified. |
| Musumeci et al. (2015) | Rheumatic diseases  Exercise | Not systematic review process – editorial article. |
| Nicholas & Rashid (2012) | MS  Any | Fatigue management was not the focus of review. |
| Pae et al. (2015) | CFS  Anti-depressants | Not systematic review process – review article. |
| Patterson et al. (2013) | Mixed  Non-pharmacological nursing interventions | Population – cancer related fatigue.  (Removed as nearly all findings related to cancer/treatment related and individual non-cancer studies either included elsewhere/found separately). |
| Payne et al. (2012) | Advanced progressive illness (mixed)  Any | Not systematic review process – only one database searched. |
| Porter et al. (2010) | CFS  Alternative medical interventions | Primary outcome of review not specifically fatigue. |
| Radbruch et al. (2007) | Palliative care  Pharmacological | Muecke et al. (2015) is full review of this protocol. |
| Reid et al. (2010) | CFS  Any | Cleare et al. (2015) update this paper. |
| Rimes and Chalder (2005) | CFS  Any | Not systematic review process – review article. |
| Rongen van Dartel et al. (2015) | RA  Aerobic exercise training | Primary outcome of review not specifically fatigue – ‘fatigue outcomes did not need to be published.’ |
| Rubin & Hotopf (2002) | Post-operative fatigue  Any | Not chronic, physical health condition – post-operative fatigue. |
| Smith et al. (2015) | CFS  Any | Primary outcome of review not specifically fatigue – fatigue grouped in with other outcomes. |
| Smith et al. (2016) | CFS  Any | Addendum to Smith et al. (2015) |
| Taylor (2006) | CFS  Rehabilitation programmes | Primary outcome of review not specifically fatigue; any outcomes reported were considered. |
| Thiem et al. (2012) | Palliative care  Glucocorticoids and androgens | Fatigue management not focus of review – conceptualised alongside wasting etc. |
| Tiesinga et al. (1999) | Mixed  Nursing interventions | Fatigue management not focus of review – assessing associated factors and identifying intervention targets. |
| Wang et al. (2014) | CFS  Traditional Chinese Medicine | Primary outcome of review not specifically fatigue; any outcomes considered.  Not systematic review process – only one database searched. |
| Whiting et al. (2001) | CFS  Any | Primary outcome of review not specifically fatigue; all outcomes were considered relevant. |
| Yee et al. (2013) | CFS  Complementary and alternative therapies | Primary outcome of review not specifically fatigue. |
| Young et al. (2014) | ALS/MND  Any | Protocol |
| Alraek et al. (2011) | CFS  Complementary and alternative medicine | Primary outcome of review not specifically fatigue. |
| Amato & Portaccio (2012) | MS  Any | Not systematic review process – general overview article |
| Bennett et al. (2016) | Cancer  Educational interventions | Cancer – related fatigue. Beyond scope of review. |
| Brenner & Piehl (2016) | MS  Pharmacological and non-pharmacological | Not systematic review process – overview article and no criteria |
| Fritschi & Fink (2012) | Type 2 Diabetes  Any | Not systematic review process – no search strategy and overview article. |
| Hadden & Hughes (2003) | Inflammatory neuropathies  Mixture | Not systematic review process – no search strategy and overview article. |
| Mehendale & Aruin (2013) | MS  Exercise approaches | Not systematic review process – overview article and inclusion criteria not clear. |
| Missaoui and Revel (2006) | Ankylosing spondylitis  Any | Not systematic review process – no search strategy and overview article. |
| Moss-Morris et al. (2016a) | MS  Exercise/behavioural | Protocol |
| Moss-Morris et al. (2016b) | MS  Exercise/behavioural | Protocol |
| Rosen et al. (2016) | Psoriasis  Biological agents | Not systematic review process – no systematic search strategy and overview article. |
| Barroso & Vos (2013) | HIV  Any | Not systematic review process – no systematic search strategy. |
| Nadarajah & Goh (2015) | Post-stroke  Pharmacological and non-pharmacological | Not systematic review process – no search strategy and review article. |
| Zhang et al. (2006) | CFS  Acupuncture | Chinese article |
| Tan et al. (n.d.) | CFS  Acupuncture | Protocol |
